# Characterization of Natural and Synthetic Sialoglycans Targeting the Hemagglutinin-Neuraminidase of Mumps Virus

**DOI:** 10.3389/fchem.2021.711346

**Published:** 2021-10-27

**Authors:** Rosa Ester Forgione, Cristina Di Carluccio, Francesco Milanesi, Marie Kubota, Ferran Fabregat Nieto, Antonio Molinaro, Takao Hashiguchi, Oscar Francesconi, Roberta Marchetti, Alba Silipo

**Affiliations:** ^1^ Department of Chemical Sciences, Complesso Universitario Monte Sant’Angelo, University of Naples Federico II, Naples, Italy; ^2^ Department of Chemistry “Ugo Schiff” and INSTM, University of Florence Polo Scientifico e Tecnologico, Florence, Italy; ^3^ Magnetic Resonance Center CERM, Sesto Fiorentino, Italy; ^4^ Department of Virology, Faculty of Medicine, Kyushu University, Fukuoka, Japan; ^5^ Laboratory of Medical Virology, Department of Virus Research, Institute for Frontier Life and Medical Sciences, Kyoto University, Kyoto, Japan

**Keywords:** mumps, NMR, molecular recognition, computational studies, inhibitors

## Abstract

The inhibition of surface viral glycoproteins offers great potential to hamper the attachment of viruses to the host cells surface and the spreading of viral infection. Mumps virus (MuV) is the etiological agent of the mumps infectious disease and causes a wide spectrum of mild to severe symptoms due to the inflammation of the salivary glands. Here we focus our attention on the hemagglutinin-neuraminidase (HN) isolated from MuV SBL-1 strain. We describe the molecular features of host sialoglycans recognition by HN protein by means of NMR, fluorescence assays and computational studies. Furthermore, we also describe the synthesis of a N-acetylneuraminic acid-derived thiotrisaccharide targeting the viral protein, and the corresponding 3D-complex. Our results provide the basis to improve the design and synthesis of potent viral hemagglutinin-neuraminidase inhibitors.

## Introduction

Mumps is an infectious disease with a high morbidity in non-immunized children, caused by a respiratory-droplets transmitted virus, known as mumps virus. Usually, it causes swollen salivary glands under the ears, referred to as parotitis; other symptoms include fever, headache, muscle aches and tiredness. Especially in adults, mumps can also cause orchitis, mastitis, pancreatitis, encephalitis and meningitis. Despite the advent of an effective vaccine, mumps virus continues to circulate throughout the world and although the majority of mumps infections are subclinical in vaccinated individuals, severe complications still occur especially in underdeveloped countries. Moreover, in the last years, mumps outbreaks among vaccinated young adults have been also reported in different countries (Willocks et al., 2017; Donahue et al., 2020).

Mumps virus is a member of the Paramyxoviridae family, belonging to the genus Orthorubulavirus, related to parainfluenza and Newcastle disease viruses, containing non-segmented negative-strand RNA genome. Different glycoproteins spikes protrude away from the viral surface, playing key roles in the different steps of the infection process as host receptor recognition, binding and cleavage. Among the 8 gene-encoded viral glycoproteins, exhibiting hemagglutinin, neuraminidase and cell fusion activity, hemagglutinin-neuraminidase (HN) has attracted attention as potential target for the development of specific and potent inhibitors able to regulate the enzyme functions. This glycoprotein is a specific sialic-acid-binding lectin able to recognize host cell-surface glycans which serve as attachment points for the virus, allowing not only its entry into the host cells but also the spreading of the infection. HN protein, indeed, also participates in the internalization of viral particles activating the fusion protein and permitting the fusion of viral membranes to the host cell. Finally, HN prevents self-agglutination of viral particles and favors the release of virions from the infected cells since it acts as a sialidase, removing the sialic acid moiety from viral progeny (Lamb and Kolakofsky, 2001). Thus, the disruption of the interactions between virus-related HN protein and host glycans may prevent the virus attachment to host cells as well as its multiplication and release from the infected cells.

With the goal to provide the basis for the design and development of novel HN inhibitors, we here report the characterization of the molecular interaction of MuV-HN protein from SBL-1 strain with α-2,3-linked sialic acid containing ligands. In detail, we described the kinetic parameters that characterize the hydrolysis of the sialyllactosamine, 3’SLN, catalyzed by the HN protein; we identified the epitope mapping of the ligand when interacting with the protein and we determined the three-dimensional structure of the protein-glycan complex. To date, the crystal structure of the MuV-HN from SBL-1 strain has not been solved yet; thus, with the aim to achieve a 3D perspective of the molecular recognition mechanism, the homology model of the protein has been built. Finally, we synthesized a potential inhibitor of HN neuraminidase and elucidated by NMR, fluorescence analysis and computational studies its interaction with the viral protein.

## Materials and Methods

### Protein Expression and Purification

Detailed method was described previously (Kubota and Hashiguchi, 2020). Briefly, the expression plasmid encoding the MuV-HN protein (amino acid positions 96–582) (Kubota et al., 2016) of the SBL-1 strain was transfected into HEK293S cells lacking *N*-acetylglucosaminyltransferase I [293S GnTI(−) cells] (Reeves et al., 2002) using polyethyleneimine-MAX (Polysciences, Inc.). MuV-HN protein was expressed as a recombinant protein containing an N-terminal secretion signal sequence and a C-terminal His_6_ tag sequence using the expression vector pHLsec (Aricescu et al., 2006; Hashiguchi et al., 2007). MuV-HN protein was first purified using a Ni^2+^-nitrilotriacetic acid (NTA) affinity column (Cosmogel His-Accept; Nacalai Tesque). Then, the eluted MuV-HN protein was further purified using a size exclusion column (Superdex 200 Increase GL 10/300; GE Healthcare) in PBS without calcium and magnesium.

### Preparation of Sialoglycans

The 3’-sialyllactosamine (3’-SLN) was purchased from Tokyo Chemical Industry Co., Lt. A thioderivative of 3’SLN, namely thio-3’SL, has been synthesized as described below.

### Synthesis and Characterization of Chemical Materials

ESI-MS analyses were performed in negative ion mode and were recorded on an LCQ-Fleet Ion Trap equipped with a standard Ionspray interface (Supplementary Figures S1, S2 in supporting information). 1H NMR spectra were obtained at 500 MHz in CDCl_3_ and D_2_O. 13C NMR spectra were obtained at 125 MHz in CDCl_3_ and D_2_O. (Supplementary Figures S3–S8 in supporting information). Chemical shifts are reported in part per million (*δ*), using the residual solvent line as internal reference. Polarimetry analysis were performed on a Jasco DIP-370.

### Synthesis of Compound 4

2 (25 mg, 0.027 mmol) and 3 (20 mg, 0.043 mmol) were solubilized in toluene and co-evaporated in vacuo for three times and then dried in vacuo overnight to remove traces of water. Under nitrogen atmosphere, anhydrous CH_2_Cl_2_ (1 ml) and freshly activated 3 Å molecular sieves (100 mg) were charged in the reaction flask and the mixture was stirred for 2 h at room temperature. The mixture was cooled to 0°C and BF_3_∙Et_2_O was added (3 μl, 0.024 mmol). The reaction was stirred at 0°C for 3 h then the mixture was diluted with 15 ml of CH_2_Cl_2_ and filtered. The filtrate was washed with 10 ml of a saturated solution of NaHCO_3_. The organic layer was collected, dried over anhydrous Na_2_SO_4_, filtered and the solvent evaporated to obtain 35 mg of crude product. Crude was purified by flash chromatography on silica gel (petroleum ether 5% in ethyl acetate; *R*_f_ = 0.35), to obtain pure 4 as a white foam (15 mg, 50%). 1H NMR (500 MHz, CDCl_3_, ∂ = 7.26): ∂ 7.37–7.22 (m, 15H, CH_2_ *Ph*); 5.64 (m, 1H, C*H*-8″); 5.29 (dd, *J*_1_ = 10.2 Hz, *J*_2_ = 2.2 Hz, 1H, C*H*-7″); 5.21 (d, *J* = 7.6 Hz, 1H, C*H*-1″); 5.10 (d, *J* = 7.5 Hz, 1H, N*H*); 5.01 (d, *J* = 11.0 Hz, 1H, C*H*_*2*_-Ph); 4.86–4.79 (m, 5H, C*H*_*2*_-Ph, C*H*-4″, C*H*-2″, C*H*_*2*_-Ph, C*H*-3″); 4.63–4.61 (m, 3H, 3xC*H*_*2*_-Ph); 4.30 (d, *J* = 7.8 Hz, C*H*-1); 4.21 (dd, *J*_1_ = 12.6 Hz, *J*_2_ = 2.6 Hz, C*H*_2_-9″); 4.09.4.02 (m, 2H, C*H*-5″, C*H*-5); 3.96–3.84 (m, 4H, C*H*_2_-9″, C*H*_2_-6, C*H*-4″, C*H*_2_-6″); 3.82 (s, 3H, COO*Me*); 3.72–3.62 (m, 5H, C*H*_2_-6’, C*H*-4, C*H*-6″, C*H*-6, C*H*-3); 3.55 (s, 3H, O*Me*); 3.54–3.51 (m, 1H, C*H*-5’); 3.36 (dd, *J*_1_ = 9.3 Hz, *J*_2_ = 8.0 Hz, 1H, C*H*-5’); 2.63 (dd, *J*_1_ = 12.8 Hz, *J*_2_ = 4.6 Hz, 1H, C*H*_*eq*_-3″); 2.17 (s, 3H, *Ac*); 2.12 (s, 3H, *Ac*); 2.03 (s, 6H, 2x*Ac*); 2.01 (s, 3H, *Ac*); 1.96 (s, 3H, *Ac*); 1.87 (s, 4H, C*H*_*ax*_-3″, *Ac*); 1.82 (s, 3H, *Ac*); 13C NMR (125 MHz, CDCl_3_, ∂ = 77.16): ∂ 171.06; 170.57; 170.45; 170.42; 170.33; 170.30; 170.24; 169.83; 169.05; 139.37; 138.88; 138.63; 128.38; 128.35; 128.16; 127.66; 127.52; 127.45; 127.43; 127.19; 104.57 (*C*H-1); 101.49 (*C*H-1″); 83.16 (*C*H-3); 82.24 (*C*H-2); 81.14 (*C*-2″); 77.54 (*C*H-4″); 75.20 (*C*H-5″); 75.08 (*C*H_2_Ph); 74.75 (*C*H_2_Ph); 73.79 (*C*H-4); 73.56 (*C*H_2_Ph); 72.46 (*C*H-5″); 69.86 (*C*H-3″); 69.59 (*C*H-6″); 69.32 (*C*H-2″); 69.11 (*C*H-4″); 67.19 (*C*H-7″); 67.15 (*C*H-8″); 62.48 (*C*H-9″); 62.16 (*C*H-6); 57.12 (O*Me*); 53.33 (COO*Me*); 49.28 (*C*H-5); 45.94 (*C*H-6″); 37.21 (*C*H-3″); 23.30 (*Ac*); 21.56 (*Ac*); 21.07 (*Ac*); 20.93 (2x*Ac*); 20.82 (*Ac*); 20.74 (*Ac*); 20.60 (*Ac*); ESI-MS m/z (%): 1264.83 (100%) [M + Na]^+^; [α]_D_^22°C^ = +46 (0.5 mg/ml in MeOH).

### Synthesis of Compound 1

To a stirred solution of 4 (11 mg 8.85 µmol) in MeOH (1 ml), Pd/C (30 mg) was added, and the suspension was stirred under H_2_ atmosphere for 24 h. Pd/C was removed by filtration on a HPLC filter and solvent evaporated to obtain 8 mg of crude as a white solid. Crude was redissolved in a freshly prepared solution of MeONa in MeOH (0.5 mg of Na° in 1 ml of MeOH) and the solution was stirred for 2 h, then solvent was evaporated, and crude dissolved in 1 ml of NaOH 1 M. The solution was stirred for 2 h, then diluted with 1 ml of water. The solution was treated with Amberlyst-15 till pH = 4, then resin was removed by filtration and the aqueous solution lyophilized to obtain pure 1 as an amorphous white solid (6 mg, 75%). 1H NMR (500 MHz, D_2_O, ∂ = 4.79): ∂ 4.55 (d, *J* = 6.9 Hz, 1H, C*H*-1″); 4.42 (d, *J* = 7.9, 1H, C*H*-1); 4.03 (dd, *J*_1_ = 12.3 Hz, *J*_2_ = 2.2 Hz, 1H, C*H*_2_-6); 3.96–3.92 (m, 1H, C*H*-8″); 3.90–3.87 (m, 4H, C*H*-4, C*H*-4″, C*H*_2_-6, C*H*_2_-9″); 3.80–3.77 (m, 1H, C*H*-5″); 3.74–3.72 (m, 2H, 2x C*H*_2_-6″); 3.71–3.64 (m, 4H, C*H*-4″, C*H*-5″, C*H*-6″, C*H*-3); 3.63–3.59 (m, 3H, C*H*-5, C*H*-7″, C*H*-9″); 3.58 (s, 3H, O*Me*); 3.43–3.46 (m, 2H, C*H*-3″, C*H*-2″); 3.32 (t, *J* = 8.65 Hz, 1H, C*H*-2); 2.82 (dd, *J*_1_ = 12.7 Hz, *J*_2_ = 4.7 Hz, 1H, C*H*_eq_-3″); 2.04 (s, 3H, *Ac*); 1.83 (t, *J* = 12.0 Hz, 1H, C*H*_ax_-3″); 13C NMR (125 MHz, D_2_O): ∂ 174.97; 174.53: 104.12 (*C*H-1″); 103.09 (*C*H-1); 84.08 (*C*-2″); 78.14 (*C*H-6″); 77.76 (*C*H-5″); 74.98 (*C*H-4″)¸74.83 (*C*H-5); 74.33 (*C*H-3); 72.82 (*C*H-2); 71.92 (*C*H-8″); 68.80 (*C*H-7″); 68.49 (*C*H-4); 68.44 (*C*H-2″); 68.11 (*C*H-4″); 62.54 (*C*H-9″); 61.24 (*C*H-6″); 60.05 (*C*H-6); 57.20 (O*Me*); 51.61 (*C*H-5″); 50.61 (*C*H-3″); 40.62 (*C*H-3″); 22.01 (*Ac*); ESI-MS m/z (%): 662.67 (100%) [M-H]^-^; [α]_D_^21^°C = +37 (0.5 mg/ml in H_2_O).

### Nuclear Magnetic Resonance Experiments

NMR spectra were collected by a Bruker 600-MHz Avance Neo instrument fitted with a cryo probe. NMR samples were dissolved in 50 mM deuterate phosphate buffer (NaCl 140 mM, Na_2_HPO_4_ 10 mM, KCl 3 mM, pH 7.4) and the [D4](trimethylsilyl)propionic acid, sodium salt (TSP, 10 uM) was used as internal reference to calibrate all the spectra. Data acquisition and processing were analyzed using TOPSPIN 3.2 software. The chemical shifts of the glycan ligands were assigned by 1H, COSY, TOCSY, NOESY and HSQC experiments (see Supplementary Tables S1, S2 and Supplementary Figures S9, S10).

1H NMR spectra were registered by using 16 k and 32 k data points. The homonuclear spectra were recorded with data sets of 4096x512 (t1 x t2) points and the data matrix processed with zero-filled in the F1 dimension up to 4096x2048 points. In order to improve the resolution, a cosine-bell function was used before Fourier transformation in both dimensions. Heteronuclear single quantum coherence (HSQC) experiments were carried out in the 1H-detected mode by single quantum coherence with proton decoupling in the 13C domain, setting data points of 2048x256. The method of States et al. (1982) was employed for the experiments in the phase-sensitive mode.

### Kinetic Analysis

For the analysis of the enzyme kinetic of 3’SLN and thio-3’SL catalyzed by MuV-HN from SBL-1 strain, a 1:70 p/l molar ratio was used. The enzyme and the substrate were dissolved in 50 mM PBS/D_2_O buffer, pH 7.4. and T 298 K. Both analyses were performed by using the following procedure: before the addition of MuV-HN of SBL-1 strain, a 1D proton spectrum with the application of composite pulses for water presaturation (zgppr) was acquired with 32 transients, thus obtaining the resonances at *t* = 0. After the addition of the ligand, followed by a short shimming routine, 1H NMR spectra using the above acquisition parameters were recorded at different time points for several hours. A timer was set to measure the delay between the addition of the protein and the collection of the first NMR spectrum of the mixture. The delay time was incorporated into the kinetic analysis of 3’SLN. The well dispersed resonances of the substrate and the products were integrated at each time point; then, the corresponding concentrations were determined with respect to the integrals of H3_eq_ resonances of the substrate. For the hydrolysis of 3’SLN by MuV-HN**,** the progress curve was fitted in Sigma Plot with the equation adapted from reference (Her et al., 2015).

### Saturation Transfer Difference Nuclear Magnetic Resonance Analysis

STD NMR spectra were acquired with a protein:ligand ratio of 1:50, with the on-resonance pulse at 6.5ppm and the off-resonance at 40 ppm. By using these conditions, no STD signals were observed in the control STD NMR spectrum of the ligand alone. A train of 50 ms (field strength of 21 Hz) Gaussian shaped pulse with an attenuation of 60 db has been used to saturate the protein. And zero-filled up to 64 k data points prior to processing. The epitope mapping of ligand 1 was achieved by the calculation of the ratio (I0–Isat)/I0, where (I0–Isat) is the intensity of the signal in the STD NMR spectrum and I0 is the peak intensity referred to the unsaturated reference spectrum (off-resonance).

### Homology Modelling and Docking Calculations

The sequence encoding for MuV-HN (UniProtKB: P19762-1, https://www.uniprot.org/) was obtained from Uniprot (http://www.uniprot.org). For computational 3D structure calculation by homology modeling, the 3D coordinates of MuV-HN head domain (PDB ID: 5B2D, chain A) were considered as template. The sequence of the target was aligned to the template using BLAST (Altschul et al., 1990) and the target-template alignment was submitted to SWISS-MODEL to generate the homology model (Waterhouse et al., 2018). Then, the obtained structure was optimized by means of Maestro suite of program (Schrödinger Suite, 2019). For the optimization, missing hydrogen atoms were added and the protonation state of ionizable groups was computed by using Maestro Protein Preparation Wizard (Schrödinger Suite, 2019). The structure was then submitted to 100,000 steps of steepest descent minimization with MacroModel and optimized with OPLS_2005 force field. The stability of the model was then evaluated by means of PROCHECK tool implemented within SAVES resource (https://saves.mbi.ucla.edu/).

The 3D coordinates of 3’-SLN and thio-3’SL were built with the help of glycan builder and Maestro (http://glycam.org, Schrödinger Suite, 2019). The bonds were parametrized and Kollman charges added by means of Maestro and the geometries of each ligand were optimized by 100,000 step of steepest descent minimization with OPLS2005 force field by using Macro Model (Schrodinger, 2012; Schrödinger Release, 2021). Ligands were prepared for docking calculations using AutoDockTools (Morris et al., 2009); docking calculations of all compounds were performed by using AutoDock 4.2.2 (Morris et al., 2009), and the analysis of the docking poses was also performed with AutoDockTools (Morris et al., 2009).

The docking protocol was validated using the crystal structure of 3’ sialyllactose ligand bound to MuV-HN (PDB-ID: 5B2D) with the aim to assess if the binding pose of the crystal was correctly predicted by docking calculations. In detail, the structure of 3’ sialyllactose was extracted from the 5B2D pdb file and docked into the binding pocket of MuV-HN from Hoshino strain. The docking parameters (grid spacing and coordinates, population size and number of energy evaluations) were optimized through this system and then employed for the docking of 3’SLN and thio-3’SL into MuV-HN SBL-1 strain model. In detail, the grid point spacing was set at 0.375 Ǻ, and a hexahedral box was built with x, y, z dimensions: 58 Ǻ, 56 Ǻ, 60 Ǻ centered in the centroid position among the active site residues Glu407, Arg422, Arg512, Tyr540 residues. A total of 200 runs using Lamarckian Genetic algorithm was performed, with a population size of 100 and 250,000 energy evaluations.

### Molecular Dynamics Simulations

MD simulations of apo-MuV-HN and MuV-HN/3’SLN complex were carried out using AMBER 18 suite of programs (Case et al., 2018).

Prior to MD simulation, the complex structure was refined upon the addition of missing hydrogen atoms and computing of protonation state of ionisable groups by means of Maestro Protein Preparation Wizard (Schrödinger Suite, 2019). Capping residues at C- and N- termini were also added using Maestro. By using the Leap module, atom types and charges were assigned according to AMBER ff14SB force field for the protein and GLYCAM-06j-1 force field to represent the ligand. The complex was hydrated with an octahedral box containing explicit TIP3P water molecules buffered at 10 Å, also, Na^+^ counter ions were added to neutralize the system by using the Leap module. The system minimization was performed using Sander and MD simulations were carried out using the CUDA, which is distributed within the AMBER 18 package.

The smooth particle mesh Ewald method was used to represent the long-range electrostatic interactions in the system while each simulation was under periodic boundary conditions, and the grid spacing was set to 1 Å. The minimization, heating and equilibration procedures prior to MD simulation were divided in several steps. The system was first minimized by applying a restriction to the protein or complex which was gradually released in the following steps. The minimization consisted of 1,000 steps of the steepest descent algorithm followed by 8,000 steps of conjugate gradient algorithm; with the application of 100 kcal·mol^−1^ A^−2^ harmonic potential as constraint that was progressively lowered before the final production. Then slow system thermalization from 0°K to 100°K was carried out applying a solute restraint of 10 kcal/mol using the Langevin thermostat in the canonical ensemble (NVT). Then, the system was heated from 100 K to 300 K in an isothermal-isobaric ensemble under the same restraint using Langevin thermostat for isothermal simulation and Berendsen barostat for constant pressure simulations (NPT). Thereafter, temperature was kept constant at 300 K during 100 ps without harmonic restraint. The systems then advanced in an isothermal-isobaric ensemble along the production run which was performed using the Langevin thermostat and Berendsen barostat, with a 2 fs time step and lasting 100 ns. The resulting trajectory was processed and analyzed (RMSD, RMSF, clustering and hydrogen bonds monitored) by means of the cpptraj module included in Amber Tools (Case et al., 2021).^.^The trajectory clustering was carried out with respect to the ligand RMSD and using the K-means algorithm (Jain et al., 1999) to acquire a total of 5 representative structures of the MD simulation.

### Fluorescence Titration

Steady-state fluorescence spectra have been collected on a Fluoromax-4 spectrofluorometer (Horiba, Edison,NJ, United States) at the fixed temperature of 10°C. Emission spectra were recorded in the emission range of 300–500 nm upon excitation at 285 nm. The slit widths were fixed at 4 nm for the excitation and 4 nm for the emission wavelength. A quartz cuvette with a path length of 1 cm and 1.5 ml volume was employed. For each experiment, MuV-HN solution at fixed concentration of 0.25 μM in PBS buffer (pH 7.4) was titrated by adding small aliquots (1–50 μl of a ligand stock solution of 700 μM) of 3’-SLN and thio-3’SL respectively. The binding curve was obtained by plotting ΔF/ΔFmax values versus ligand concentration as described by Ribeiro et al. (2008).

## Results

Receptor motifs that can be recognized by MuV-HN (hemagglutinin-neuraminidase) protein from SBL-1 strain have been recently identified by glycan array screening (Kubota et al., 2019) as specific host-cell sialoglycans which terminate with neuraminic acid (Neu5Ac) moiety. Among them, Neu5Acα2-3Galβ1-4GlcNAc (3’SLN), a sialoglycan broadly expressed in and exposed on various host tissues, was reported as one of the strongest binders; thus, we decided to investigate, at molecular level, its recognition by MuV-HN SBL-1 strain by means of NMR spectroscopy, biophysical and computational approaches.

**FIGURE 1:**
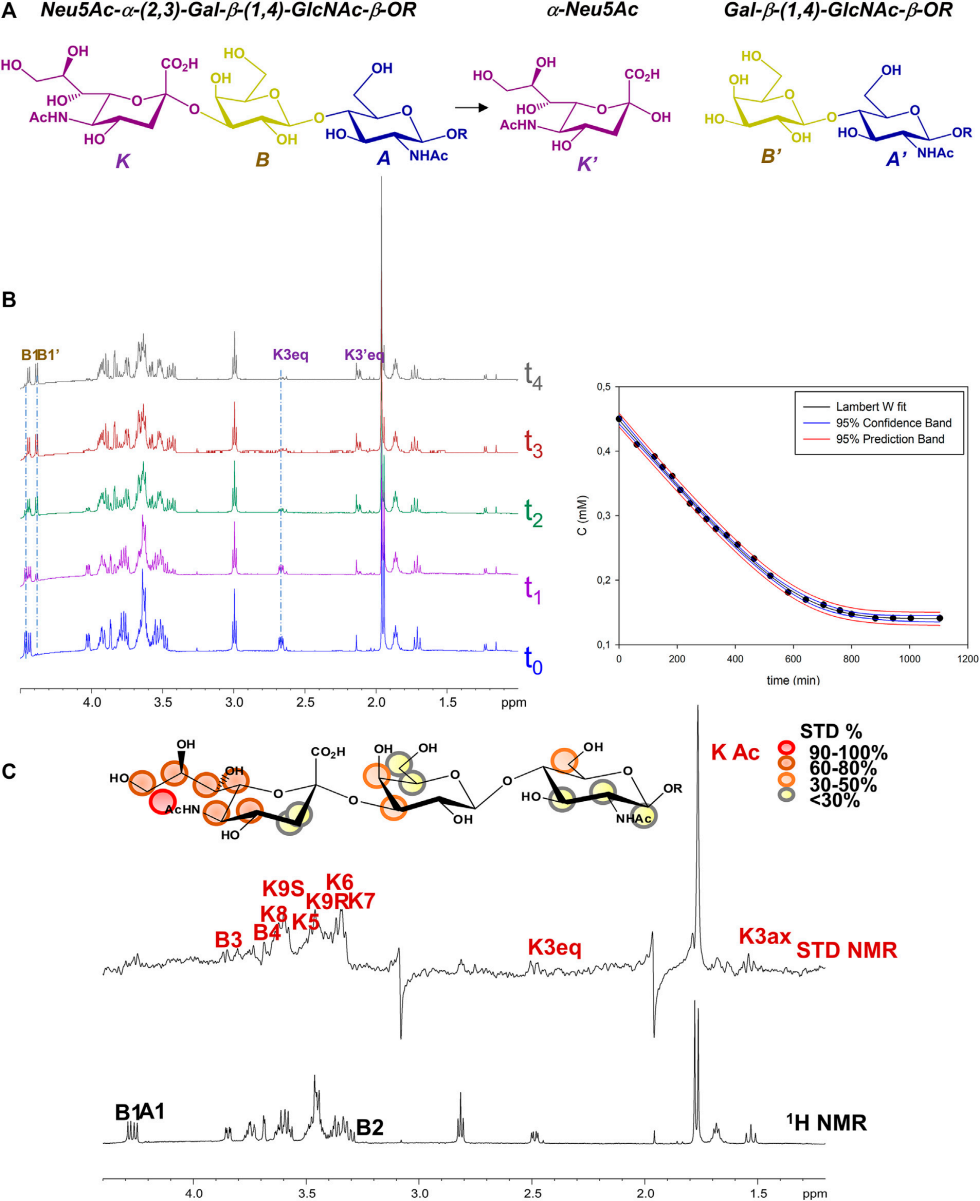
NMR analysis of MuV-HN from SBL-1 in complex with 3’SLN **(A)** Scheme of the mechanism of hydrolysis of 3’SLN catalyzed by MuV-HN (SBL-1 strain). **(B) (left panel)** Section of 1H-NMR spectra at different time of the enzymatic reaction have been reported. The spectra were recorded using 13 μM of MuV-HN protein and 700 μM of 3’SLN in PBS deuterate buffer (pH = 7). The NMR quantification of the substrate concentration was performed by integration of the well-dispersed resonances of the product (B1, K3_eq_). **(right panel)** Analysis of the MuV-HN kinetics toward 3’SLN by means of the explicit reformulation of the integrated form of MM equation with Lambert W fit as solution. The plot of the concentration of 3’SLN as a function of the time has been reported. The substrate concentration was evaluated from the K3_eq_ resonance of Neu5Ac unit. The fit of the kinetic data by the Lambert-W fit produced a K_M_ value of 5 μM and V_max_ of 6*10^−3^ (mM/min). The blue dashed line represented the confidence interval of the fit and the red dashed lines are the prediction bands. **(C)** STD NMR analysis of 3’SLN in the interaction with MuV-HN from SBL-1 strain. **(top panel)** Interacting epitope map of 3’SLN as derived by STD-NMR data. **(bottom panel)** The 1H NMR spectrum and the STD NMR spectrum of MuV-SBL-1–3’SLN mixture with a molecular ratio of 1:70, at 283 K.

Given the enzymatic activity of MuV-HN SBL-1, upon addition of the sialoglycan substrate in solution, MuV-HN protein catalyzed the cleavage of the terminal Neu5Ac moiety, producing *N*-Acetyllactosammine (LacNAc, residues A’ and B’) and reducing sialic acid (*red-*Neu5Ac, K’) (Figure 1A). Therefore, the real time kinetics of 3’SLN hydrolysis by MuV-HN was followed by 1D 1H-NMR, as reported in Figure 1B, where the overlapped NMR spectra detected over time progression are shown. The progress of the hydrolysis was assessed by monitoring the decrease of the signals of the substrate (as B1 and K3_eq_) and the concurrent appearance and following enhancement of the intensity of the products resonances (as B’1 and K’3_eq_). Similarly to other viral sialidases, (Chan et al., 2012), MuV-HN acts as retaining glycosidase, with a net retention of substrates configuration. The H3_eq_ resonance of Neu5Ac was monitored to evaluate the variation of the substrate concentration and, by fitting the kinetic data through the Lambert W function (Goudar et al., 2004), the values of the enzyme K_M_ value of 5 μM and V_max_ of 6*10^−3^ mM/min were determined (Figure 1B). The low value of the K_M_ corresponds to high affinity for 3’SLN substrate.

Notably, the K_M_ evaluated for 3’SLN/MuV-HN from SBL-1 strain was lower with respect to that observed by our group for the same substrate in the interplay with MuV-HN from Hoshino strain. (K_M_ 12 μΜ) (Forgione et al., 2020), suggesting a higher enzymatic affinity of the protein from SBL-1 strain for 3’SLN.

Despite the neuraminidase activity of MuV-HN induces the cleavage of the sialic acid within NMR time scale, saturation transfer difference (STD) NMR allowed to identify the ligand binding epitope (Figure 1C) (Di Carluccio et al., 2021). A qualitative analysis of the STD effects demonstrated that the trisaccharide was entirely accommodated into the binding pocket of the protein. The moiety most contributing to the interaction was the neuraminic acid residue (K) which established several contacts with the protein, not only through its acetamide portion but also via the lateral chain; the highest STD effect, set at 100%, belonged indeed to the N-acetyl group; protons H7–H8–H9 also showed significant STD enhancements; finally, STD signals were detected for some protons of the Neu5Ac carbohydrate ring, as H4, H5 and H6. As already observed in the case of MuV-HN from Hoshino strain, also the other two residues (A and B) were involved in the interaction with the protein although to a lower extent. Furthermore, by evaluating the relative intensities of two N-acetyl groups of Neu5Ac K and GlcNAc A, it was clear that the contribution from the acetamide of the GlcNAc was not significant.

**FIGURE 2:**
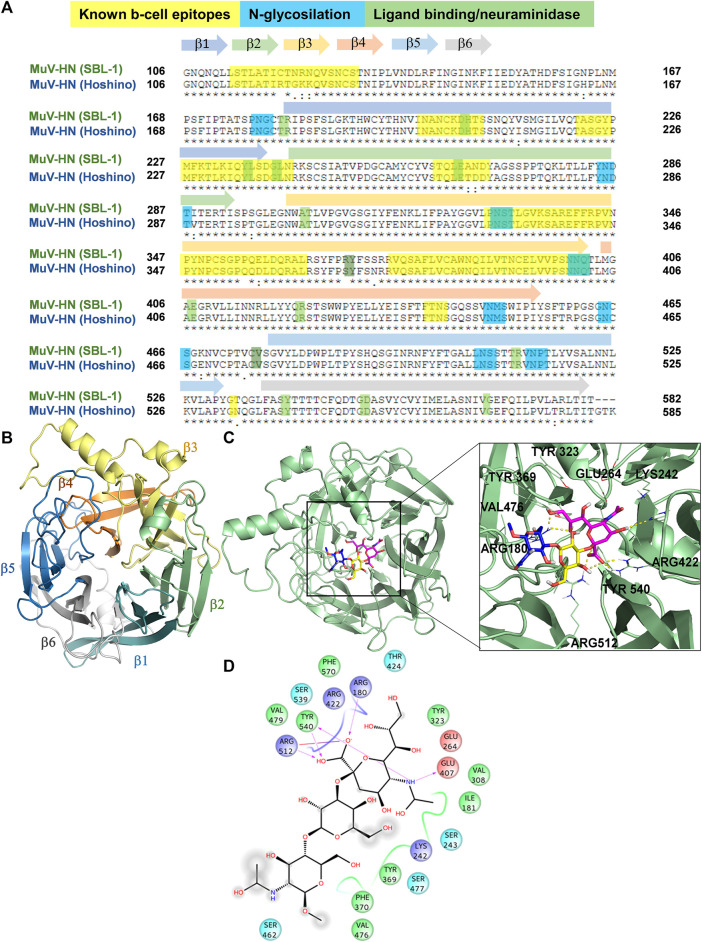
3D model of MuV-HN from SBL-1 in complex with 3’SLN **(A)** CLUSTAL alignment of the head domain of MuV-HN from different strains. Amino acids involved in molecular recognition and neuraminidase activity are highlighted in green, N-glycosylation sites are highlighted in blue and Known B cell epitopes in yellow. Sequences corresponding to the six four-stranded beta sheets are indicated with differently colored arrows. **(B)** MuV-HN monomer six bladed propeller structure, whose constituent structural elements (β1–β6 sheets) are differently colored. **(C)** 3D model of MuV-HN from SBL-1 in complex with 3’SLN. Close up view of 3’SLN binding pose at the MuV-HN active site. The main amino acid residues involved in the binding are highlighted as lines. Sialic acid, Galactose and N-acetyl Glucosamine residues are colored in magenta, yellow and blue, respectively according to the SNFG nomenclature. **(D)** Two-dimensional plot illustrating the interactions of 3’SLN with the MuV-HN binding site residues. Dotted arrows indicate hydrogen bonds with functional groups from side chains and solid arrows those involving the backbone functional groups. The residues shown, close to the ligand, are involved into hydrophobic and polar interactions.

To get structural insights into sialoglycans recognition by MuV-HN (SBL-1 strain, genotype A), comparative homology modelling was carried out considering as template the crystal structure of MuV-HN head domain (Hoshino strain, genotype B), PDB ID: 5B2D, which possesses 95% of structural similarity to the target sequence (Figure 2A), in agreement with the low number of genomic mutations occurring among different MuV genotypes at HN encoding gene (Palacios et al., 2005). From the sequence analysis of the two different strains (Figures 2A,B and Supplementary Figure S11), the most relevant modifications of the amino acid sequences occurred at the known B-cell epitopes, particularly at 327–363; 375–403; 440–443; and 533 regions, as similarly detected when comparing other MuV-HN genotypes (Gouma et al., 2020).

The homology model of HN protein from SBL-1 strain was then built using Swiss Model server (Waterhouse et al., 2018), and, after refinement by means of Maestro (Schrödinger Suite, 2019), its quality was assessed through SAVES resource (https://saves.mbi.ucla.edu/). As result, the refined model, analyzed using ERRAT plot, exhibited an overall quality factor of 87.11. The Ramachandran plots generated by means of the PROCHECK tool (Supplementary Figure S11B) allowed to assess the stereochemical quality of the resulting structure (Laskowski et al., 1993), showing that 99% of the residues’ dihedrals belonged to the core/allowed regions, hence confirming the fitness of the model. Moreover, the 3D profile of the model structure obtained by VERIFY 3D, scoring the compatibility of the 3D structure model with respect to amino acid sequence, showed a scoring above 0.2 for above 80% of the amino acid residues, further confirming the accuracy of the model (Supplementary Figure S11C). Comparing our refined model with the target structure, a similar topology was noted, and the RMSD calculation in reference to the MuV-HN crystal structure of 0.05 Å further denoted its remarkable quality, in full agreement with the high sequence identity between the homologous proteins from different MuV strains (Supplementary Figure S11A). Indeed, as predicted by the sequence analysis, the changes in the amino acid sequence among the proteins from the two different strains did not affect neither the overall tridimensional structure of the sialic acid binding motif nor the accessibility of the protein to the glycans on the host cell surface. In detail, MuV-HN (SBL-1 strain) model showed a globular structure, characterized by six four-stranded antiparallel β−sheets (β1-β6) arranged in the so-called bladed beta propeller fold, that is characteristic of many viral neuraminidases (Colman et al., 1993) (Figure 2B). At the center of the domain, it was located the dual binding/sialic acid cleavage site of the protein, comprising the conserved sialidase active site residues, namely Arg180, Glu407, Arg422, Arg512, Tyr540, Glu561, and Asp204 (Kubota et al., 2016). Furthermore, the binding site comprised the Tyr369 and Val476, highly conserved among the MuV-HN genotypes, that revealed to be important determinants of Mumps recognition (Kubota et al., 2016; Forgione et al., 2020; Gouma et al., 2020).

As several studies highlighted the importance of the flexibility of specific loops in other viral sialidases (Winger et al., 2012; Kubota et al., 2019), the MuV-HN conformational behavior was explored by MD simulations. First, the MD simulation of the apo form of MuV-HN SBL-1 strain model was carried out using Amber18 (Case et al., 2018). As result, it was noted that MuV-HN showed a slight conformational rearrangement along the simulated time, with a small increase of the backbone RMSD which converged to 1.5 Å (Supplementary Figure S12A). The inter-strand loops RMSD was calculated (Supplementary Figure S12B) showing that the fluctuations from the initial geometry affected mostly β5 and β6 loops. No relevant conformational changes were instead detected when calculating the β1, β2 and β3 inter-strand loops RMSD along the trajectory (Supplementary Figure S12B). Furthermore, the atom-positional root-mean-square fluctuations (RMSFs) relative to the Cα-atoms was calculated along the trajectory, denoting smaller values in the secondary structure regions, further reinforcing the homology model quality (Supplementary Figure S12C). Interestingly, large fluctuations occurred in the loop regions, especially at the positions corresponding to the β4 and β6 inter-strand loops. In particular, the β4 showed high flexibility from the RMSF plot (Supplementary Figure S12B). Nonetheless, the highest flexibility belonged to the long loop connecting β4 and β5 sheets.

Our model was subsequently employed for docking calculation by Autodock 4.2 (Morris et al., 2009) program in the presence of 3’SLN as ligand. Based on the predicted binding energy and cluster population (Supplementary Table S1), the best pose of 3’SLN/MuV-HN complex was selected (Figures 2C,D) and it was submitted to 100 ns MD run using AMBER 18 (Supplementary Figures S13A–E). The stability of the complex has been assessed by the ligand RMSD calculated using the protein as reference; the results showed that the ligand remained stable around 1 Å for almost the entire simulation, thus indicating it was anchored to the receptor along the simulated time. Moreover, the notable structural similarity between the most populated MD clusters calculated with respect to the ligand RMSD along the trajectory further confirmed that the sialotrisaccharide didn’t exhibited conformational changes along the simulation (Supplementary Figures S13A,B).

In accordance with NMR data, computational analysis showed that all three sugar moieties were in contact with the receptor surface, as reported in Figure 2C. The interaction network of the selected complex showed many similarities with those observed for MuV-HN from Hoshino strain (Kubota et al., 2016; Kubota et al., 2019; Forgione et al., 2020). In detail, the sialic acid residue established strong interactions between its carboxylate moiety and three arginine residues, namely Arg180, Arg422 and Arg512. The N-Acetyl moiety formed the hydrogen bond with Glu407 and hydrophobic interactions with Ile181 and Val308. The GlcNAc ring was engaged in CH-pi interactions with Tyr369. Gal residue was in proximity to the receptor surface but no significant polar interactions were observed, as its hydroxyl groups were directed toward the solvent. Nonetheless, the Gal moiety was in proximity to the Val476 side chain thus entailing hydrophobic contacts with MuV-HN. Additionally, the analysis of the protein-ligand interactions showed that the main contacts were maintained along all the trajectory, including the electrostatic interactions of the Neu5Ac unit with the Arg triad, and the stacking interaction between Tyr369 and the GlcNAc moiety (Supplementary Figures S13C–E). This further suggested the importance of such residues to the molecular recognition of sialoglycans by Mumps virus hemagglutinin-neuraminidase receptor.

In order to verify if a particular loop stabilization occurred in the MuV-HN bound form, the RMSF and loop RMSD analysis of the 3’SLN/MuV-HN complex was carried out and compared to the free state. As a result (Supplementary Figures S14A,B), lower fluctuations upon binding of the inter-strands loops of β5 and β6 sheets were observed. A significant stabilization of the inter-sheet loops, namely β2-β3, β4-β5 and β5-β6 was also noted. Interestingly, the lower atomic fluctuations displayed by the loops of β4, β5 and β6 sheets matched with the MuV-HN region binding to the 3’SLN ligand, therefore implying that the stabilizing interactions with the sialotrisaccharide influenced the involved loops conformational flexibility.

Given that from NMR and computational analysis the entire trisaccharide seemed to be involved in the recognition and binding process, being the sialic acid the moiety anchoring the glycan to the binding pocket of the protein, we decided to synthesize a thioderivative of 3’SLN, namely thio-3’SL, as a potential HN inhibitor. The incorporation of a sulfur functional group on the anomeric position of the sialic acid should indeed affect the lability of the Sia-Gal glycosidic linkage without hampering the recognition of the sialoglycan by the enzyme.

**SCHEME 1:**
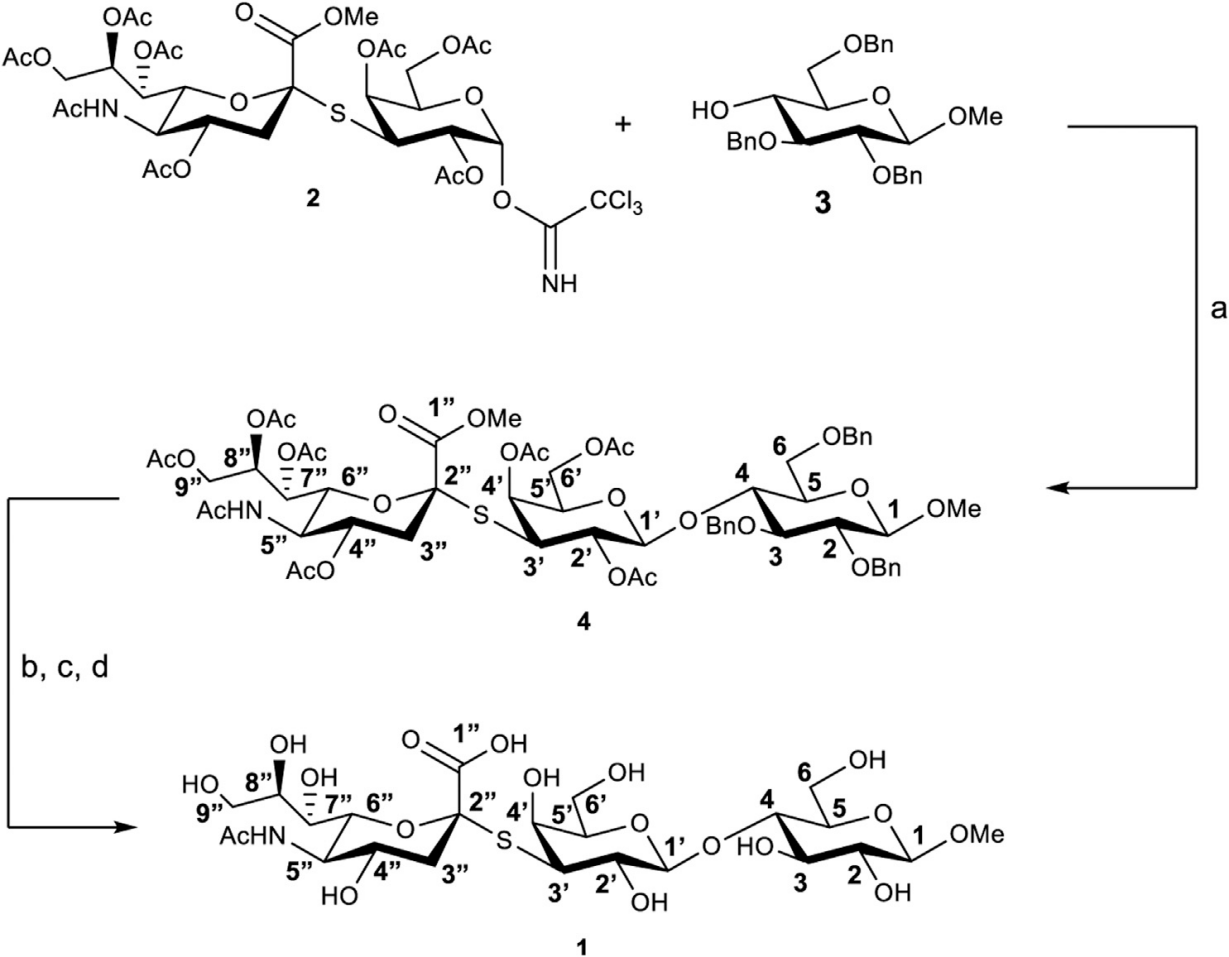
Synthesis of thio-3’SL (compound 1) with atoms labelling. Reagents and conditions: **(A)** BF_3_∙Et_2_O, 3Å molecular sieves, dry CH_2_Cl_2_, 0°C, 3 h; **(B)** Pd/C, H_2_, MeOH, room temperature, 24 h; **(C)** MeONa, MeOH, room temperature, 2 h; **(D)** NaOH 1M in H_2_O, room temperature, 2 h.

The thio-3’SL (1) was prepared (see Scheme 1) starting from the thrichloroacetimidate 2, (Turnbull et al., 2000), and the methyl-2,3,6-tri-O-benzyl-β-D-glucopyranoside (3), (Yoneda et al., 2005), both prepared accordingly to literature methods. Glycosylation, catalyzed by BF_3_∙Et_2_O, gives the protected trisaccharide 4 with a 50% yield. Removal of benzyl protective groups by hydrogenation and of the acetylic groups with MeONa in MeOH was followed by hydrolysis of the methyl ester with aqueous NaOH to give the thiotrisaccharide 1 with a 75% yield on three steps.

**FIGURE 3:**
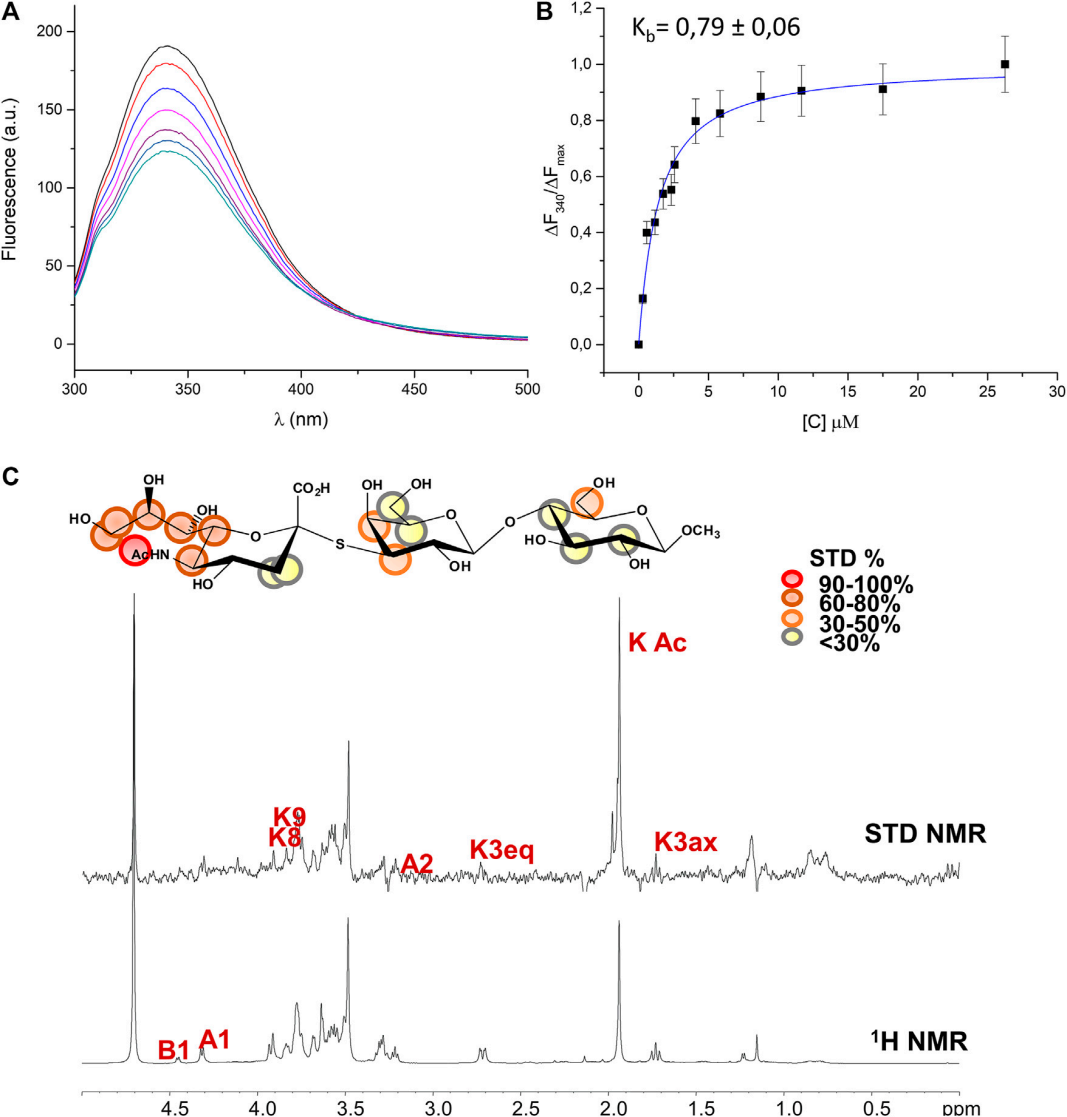
Analysis of MuV-HN from SBL-1 in complex with thio-3’SL **(A)** Fluorescence titration of MuV-HN (0.25 μM) upon the addition of increasing concentration of thio-3’SL from a stock solution of 700 μM. The emission spectra were recorded by using an excitation wavelength of 285 nm and a temperature of 10°C. The relative binding isotherm, the value of the binding constant (K_b_) is also reported. For each data point, 10% Y error bars are shown. **(B)** STD NMR analysis of thio- 3’SL in the interaction with MuV-HN from SBL-1 strain. **(top panel)** Interacting epitope map of thio 3’SL as derived by STD-NMR data. **(bottom panel)** The 1H-NMR spectrum and the STD NMR spectrum of MuV-SBL-1–thio-3’SL mixture with a molecular ratio of 1:50, at 283 K.

The hydrolytic stability of the thio-3’SL and its ability to interact with the MuV-HN protein was hence investigated by means of NMR spectroscopy and fluorescence analysis. As expected, the thio-3’SL was not hydrolyzed by MuV-HN, as it was observed by monitoring over time the NMR spectra of MuV-HN/thio-3’SL mixture (data not shown). However, it was recognized by MuV-HN protein as suggested by fluorescence data. The binding interaction of thio-3’SL with MuV-HN from SBL-1 strain was indeed analyzed by fluorescence spectroscopy exploiting the presence of aromatic amino acids in the protein binding pocket. In detail, changes in the fluorescence intensity of tryptophane residues of MuV-HN upon the addition of increased amount of thio-3’SL were followed (Figure 3A left panel). The interaction was quantitatively evaluated by fitting the resulting binding curve achieved with the application of the quenching data elaboration described by Ribeiro et al. (Ribeiro et al., 2008). As a result, a binding constant (K_b_) value of 0.44 ± 0.05 was obtained. (Figure 3A, right panel).

**FIGURE 4:**
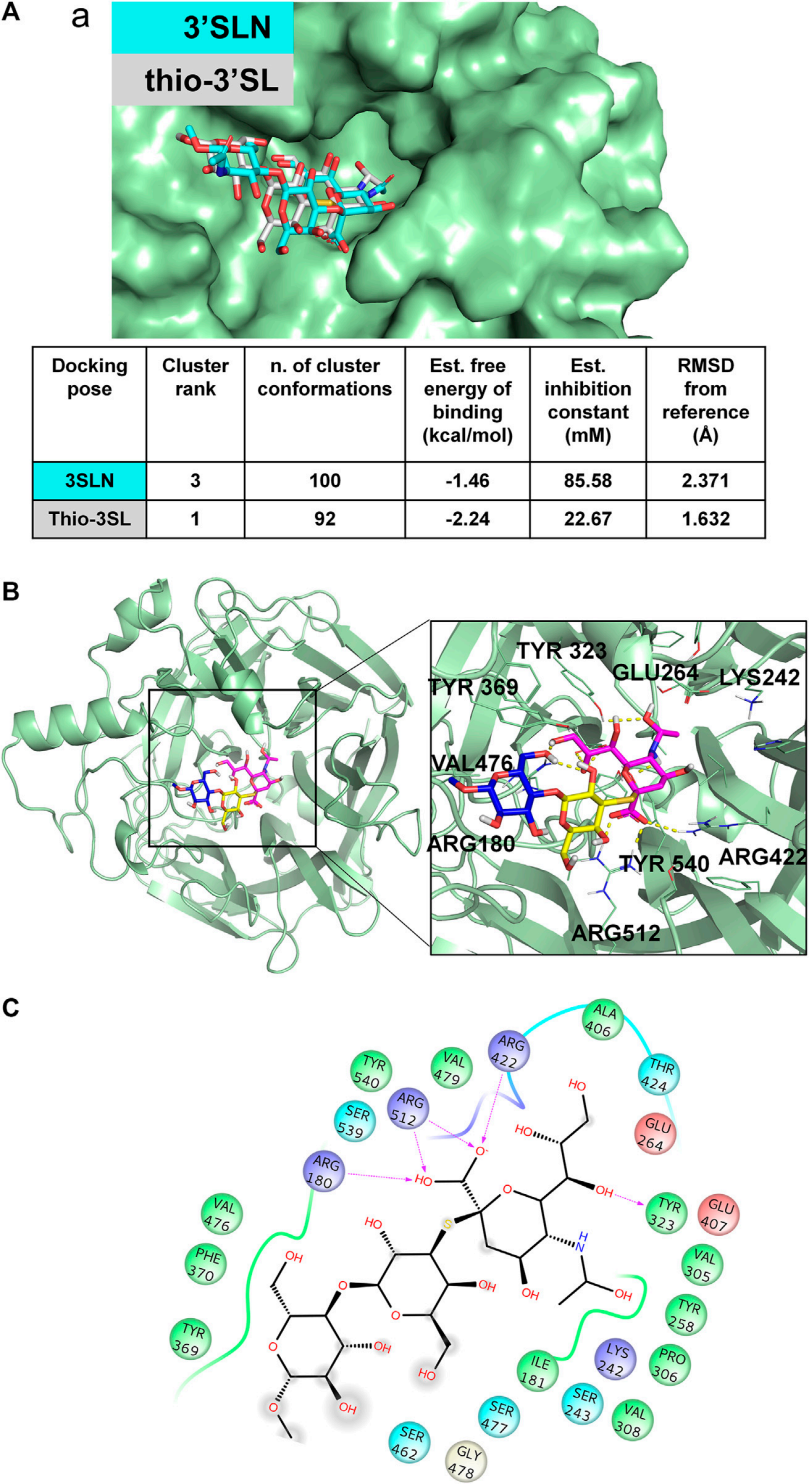
3D model of MuV-HN from SBL-1 in complex with thio-3’SL **(A)** Docking of 3’SLN and thio-3’SLN into MuV-HN from SBL-1 strain structural model. The superimposition of the best selected binding mode of both ligands is shown. The parameters of the best models, calculated by Autodock, are also enlisted. **(B)** Close up view of thio-3’SL binding pose at the MuV-HN active site. The main amino acid residues involved in the binding are indicated as lines. Sialic acid, Galactose and N-acetyl Glucosamine residues are colored in magenta, yellow and blue, respectively according to the SNFG nomenclature. **(C)** Two-dimensional plot showing the interactions of thio-3’SL with the MuV-HN binding site residues. The residues shown are involved into hydrophobic and polar interactions with the ligand.

The molecular characterization of the complex has been then carried out by the combination of NMR and computational studies. The ligand interacting epitope has been indeed described by STD NMR experiments (Figure 3B). As expected, the strongest STD enhancements were observed for the sialic acid moiety and especially for its acetyl group and protons of Neu5Ac lateral chain (H7-H8-H9). Significant STD effects were further detected for protons H4-H5 and H6 of sialic acid ring. Lower STD signals (below 50%) were instead observed for protons belonging to galactose and glucose residues indicating that they pointed farer from the protein surface. The thio-3’SL structure was then modelled into the binding site of MuV-HN SBL-1. The docking calculations allowed to describe the relevant receptor-ligand binding interactions, highlighting that the protein accommodated the synthetic analogue and the 3’SLN with a similar binding mode. Indeed, the superimposition of the two complexes (Figure 4A) clearly showed a nearly identical orientation of the ligands into the receptor binding site. The interaction network was overall conserved; the Neu5Ac carboxylate engaged electrostatic interactions with the receptor Arg180, Arg422 and Arg512; Glu407, Glu264, and Tyr323 binding pocket residues were also interacting with sialic acid unit. Moreover, the Glc moiety of the trisaccharide formed hydrophobic interactions with Tyr369 and Val476 residues; this latter residue was also in close contact with Gal moiety.

## Discussion

Hemagglutinin-Neuraminidase activity is essential for the infection and propagation of viruses belonging to the Paramyxoviridae family, including parainfluenza (hPIV), the Newcastle disease and mumps viruses. Over the past years, different series of inhibitors have been developed toward viral neuraminidases, especially targeting hPIVs. However, the design and development of novel inhibitors and high-affinity ligands could prove worthwhile for a better understanding of virus tropism and pathogenesis and may help in the fight against viral infections allowing the advancement of new licensed anti-viral drugs.

Here we focused our attention on mumps virus, the leading cause of the mumps disease that could affect the central nervous system, causing meningitis. Mumps virus possesses different surface glycoproteins and among them, the HN represents a multitasking protein mediating both the early and late stages of viral infection, including the host-cell sialoglycans recognition, the trigger of virus and host-cell membranes fusion and finally the release of progeny virions from infected cells. MuV-HN thus represents an attractive target for the structure-based design and development of novel anti-viral drugs since interfering with these processes may affect the viral pathogenicity and infectivity.

Thus, we investigated the structural features of MuV-HN from SBL-1 strain when bound to host-cell sialoglycans with the aim to exploit them as basis for the guided development of tailored inhibitors. The structural characterization of the HN protein in the interaction with the 3’SLN has been performed by means of NMR and computational studies. In detail, the kinetic of 3’SLN hydrolysis catalyzed by MuV-HN from SBL-1 strain was characterized by progress curve analysis derived by NMR assay. Notably, the kinetic parameters obtained by the elaboration through the Lambert W function were in the same range of the values obtained for the same ligand interacting with MuV-HN from Hoshino strain, as shown in our previous work (Forgione et al., 2020). STD NMR allowed to describe the ligand interacting epitope stressing the importance of the sialic acid in the recognition and interaction process, as expected, but also demonstrating that the binding pocket of the protein was pliable enough to accommodate the three composing sugars of the 3’SLN.

**FIGURE 5:**
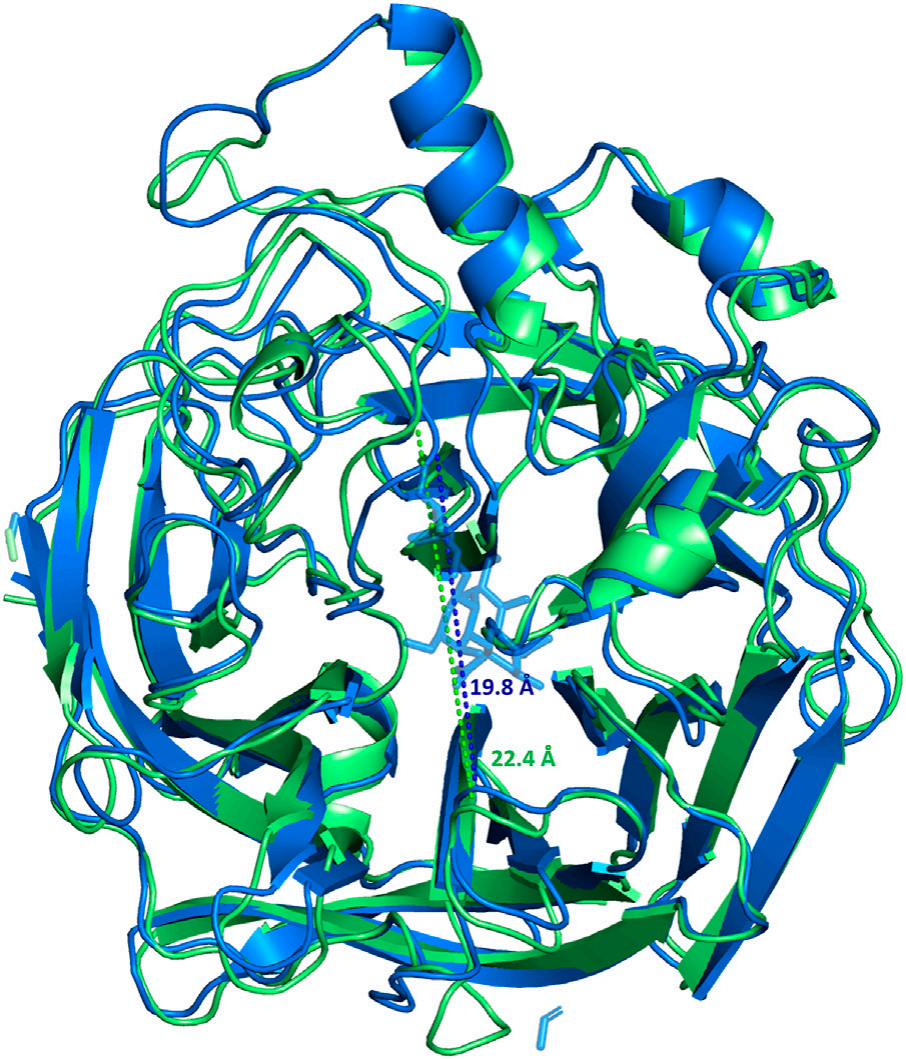
Superimposition of the most populated clusters of MuV-HN from SBL-1 strain in complex with 3’SLN ligand (blue) and the apo-MuV-HN (green), extracted from the MD simulations. The distances between the walls of the binding site are 19.8 Å (blue dashed line) and 22.4 Å (green dashed line) for the complexed and apo forms, respectively.

The homology modelling of the protein was also carried out with the aim to achieve a 3D view of the protein-ligand complex. The crystal structure of MuV-HN from the Hoshino strain was used for building the model; that revealed a huge similarity between the 3D structures. It is worth to note that, from the sequence analysis of the two proteins, few variable positions were observed. Few of the mutations affected the important functional regions, as the known B-cell epitopes; the most relevant is the 354 (P→Q) mutation at the 327–363 region, that modifies the physiochemical features of the surface exposed area. This variation was already suggested to be significant for altered antibody recognition and neutralization by MuV-HN (Gouma et al., 2018; Gouma et al., 2020). Notably, the neuraminidase binding site was preserved, differently with respect to that observed when comparing the sequences of other MuV-HN genotypes (Gouma et al., 2018; Gouma et al., 2020). The refined 3D model of the protein has been used for docking analysis and MD simulations of the MuV-HN/3’SLN complex. Our results demonstrated that the main interactions involving the three sugars of the glycan receptor observed when bound to MuV-HN from Hoshino strain were completely conserved in the binding with MuV-HN from SBL-1 strain. We indeed observed that the Neu5Ac residue was deeply located in the receptor pocket where it established strong polar interactions with the canonical neuraminidases sialic acid recognizing residues, including the Arginine triad (Arg180, Arg422, Arg512). Nonetheless, the previously described interactions involving Tyr369, which were reported to strongly influence the binding affinity of MuV-HN from Hoshino strain, were also present in the case of MuV-HN from SBL-1 strain (Kubota et al., 2016). Interestingly, the MD simulation studies performed on the apo and bound forms of MuV-HN revealed the stabilization of particular inter- strand- and inter-sheet loops upon the ligand binding, thus suggesting that the receptor loop flexibility may play a relevant role in the molecular recognition of sialoglycans by mumps virus hemagglutinin neuraminidase and mechanism of catalysis as well. Indeed, in the bound state, lower fluctuations were observed for loop regions in intimate contact with the ligand bound to Muv-HN, on which some of the binding site residues were located, as Val476 on β4-β5 loop and Arg512 on β5 loop. Previous studies on the conformational behavior of other neuraminidases (Winger et al., 2012; Amaro et al., 2009; Amaro et al., 2011) reported a shift from open to closed loop conformation upon sialoglycans binding, thus, we also inspected this possibility by measuring the distance across the loops delimiting the binding site (Figure 5). Interestingly, the distance measured between the binding pocket walls showed an increased value for the apo-MuV-HN protein, thus suggesting that also for the mumps virus hemagglutinin neuraminidase the glycan receptor specificity may be tuned by loops flexibility.

Furthermore, we performed the synthesis of a thiotrisaccharide, thio-3’SL, as a potential inhibitor of the neuraminidase activity of the protein. The ability of thio-3’SL to interact with MuV-HN from SBL-1 strain was demonstrated by monitoring the intrinsic fluorescence of the tryptophane residues of the receptor and by NMR. Moreover, modelling analyses provided a picture of the protein-ligand complex, showing that the thio-3’SL established contacts with MuV-HN receptor which were comparable to those made from 3’SLN, consistently to the similar bound ligands’s topology. The main interactions mostly involved the Neu5Ac unit, that interacted with the characteristic receptor binding site amino acids Arg180, Arg422, Arg512, Glu407 and Tyr323. Accordingly, the third sugar exhibited stacking interactions with the Tyr369.

In conclusion, a combination of organic chemistry, NMR, fluorescence and computational studies could provide a 3D view of the MuV-HN from SBL-1 strain when interacting with the natural substrate, 3’SLN, and the synthetic thio-3’SL, showing a similar binding mode. Our analysis demonstrated that, in both cases, the main interactions involving the three sugars of the glycan receptor were also comparable to those observed when investigating MuV-HN from Hoshino strain (Kubota et al., 2016).

Our outcomes may help in the identification of new inhibitor scaffolds which could prove worthwhile in the fight against mumps virus.

## Data Availability

The original contributions presented in the study are included in the article/Supplementary Material, further inquiries can be directed to the corresponding author.

## References

[B1] Altschul S. F. Gish W. Miller W. Myers E. W. Lipman D. J. (1990). Basic Local Alignment Search Tool. J. Mol. Biol. 215 (3), 403–410. 10.1016/S0022-2836(05)80360-2 2231712

[B2] Amaro R. E. Cheng X. Ivanov I. Xu D. McCammon J. A. (2009). Characterizing Loop Dynamics and Ligand Recognition in Human- and Avian-type Influenza Neuraminidases via Generalized Born Molecular Dynamics and End-point Free Energy Calculations. J. Am. Chem. Soc. 131 (13), 4702–4709. 10.1021/ja8085643 19296611 PMC2665887

[B3] Amaro R. E. Swift R. V. Votapka L. Li W. W. Walker R. C. Bush R. M. (2011). Mechanism of 150-cavity Formation in Influenza Neuraminidase. Nat. Commun. 2, 388. 10.1038/ncomms1390 21750542 PMC3144582

[B4] Aricescu A. R. Assenberg R. Bill R. M. Busso D. Chang V. T. Davis S. J. (2006). Eukaryotic Expression: Developments for Structural Proteomics. Acta Crystallogr. D Biol. Cryst. 62, 1114–1124. 10.1107/S0907444906029805 17001089 PMC7161643

[B5] Case D. A. Cerutti D. S. Cheatham T. Darden T. Duke R. E. Gieses T. J. (2018). AMBER 2018. San Francisco, CA: University of California.

[B31] Case D. A. Aktulga H. M. Belfon K. Ben-Shalom I. Y. Brozell S. R. Cerutti D. S. (2021). AMBER 2021. San Francisco, CA: University of California.

[B6] Chan J. Watson J. N. Lu A. Cerda V. C. Borgford T. J. Bennet A. J. (2012). Bacterial and Viral Sialidases: Contribution of the Conserved Active Site Glutamate to Catalysis. Biochemistry 51 (1), 433–441. 10.1021/bi201019n 22133027

[B7] Colman P. M. Hoyne P. A. Lawrence M. C. (1993). Sequence and Structure Alignment of Paramyxovirus Hemagglutinin-Neuraminidase with Influenza Virus Neuraminidase. J. Virol. 67 (6), 2972–2980. 10.1128/JVI.67.6.2972-2980.1993 8497041 PMC237633

[B8] Di Carluccio C. Forgione M. C. Martini S. Berti F. Molinaro A. Marchetti R. (2021). Investigation of Protein-Ligand Complexes by Ligand-Based NMR Methods. Carbohydr. Res. 503, 108313. 10.1016/j.carres.2021.108313 33865181

[B9] Donahue M. Hendrickson B. Julian D. Hill N. Rother J. Koirala S. (2020). Multistate Mumps Outbreak Originating from Asymptomatic Transmission at a Nebraska Wedding - Six States, August-October 2019. MMWR Morb. Mortal. Wkly. Rep. 69 (22), 666–669. 10.15585/mmwr.mm6922a2 32497030 PMC7272110

[B10] Forgione R. E. Di Carluccio C. Kubota M. Manabe Y. Fukase K. Molinaro A. (2020). Structural Basis for Glycan-Receptor Binding by Mumps Virus Hemagglutinin-Neuraminidase. Sci. Rep. 10 (1), 1589. 10.1038/s41598-020-58559-6 32005959 PMC6994497

[B11] Goudar C. T. Harris S. K. McInerney M. J. Suflita J. M. (2004). Progress Curve Analysis for Enzyme and Microbial Kinetic Reactions Using Explicit Solutions Based on the Lambert W Function. J. Microbiol. Methods. 59 (3), 317–326. 10.1016/j.mimet.2004.06.013 15488275

[B12] Gouma S. Vermeire T. Van Gucht S. Martens L. Hutse V. Cremer J. (2018). Differences in Antigenic Sites and Other Functional Regions between Genotype A and G Mumps Virus Surface Proteins. Sci. Rep. 8 (1), 13337. 10.1038/s41598-018-31630-z 30190529 PMC6127219

[B13] Gouma S. Vermeire T. Van Gucht S. Martens L. Hutse V. Cremer J. (2020). Publisher Correction: Differences in Antigenic Sites and Other Functional Regions between Genotype A and G Mumps Virus Surface Proteins. Sci. Rep. 10 (1), 5456. 10.1038/s41598-020-62301-7 32198462 PMC7083855

[B14] Hashiguchi T. Kajikawa M. Maita N. Takeda M. Kuroki K. Sasaki K. (2007). Crystal Structure of Measles Virus Hemagglutinin Provides Insight into Effective Vaccines. Proc. Natl. Acad. Sci. 104, 19535–19540. 10.1073/pnas.0707830104 18003910 PMC2148324

[B15] Her C. Alonzo A. P. Vang J. Y. Torres E. Krishnan V. V. (2015). Real-Time Enzyme Kinetics by Quantitative NMR Spectroscopy and Determination of the Michaelis-Menten Constant Using the Lambert-W Function. J. Chem. Educ. 92 (11), 1943–1948. 10.1021/acs.jchemed.5b00136

[B35] Jain A. K. Murty M. N. Flynn P. J. (1999). Data Clustering: A Review. ACM Computing Surveys 31, 264–323. 10.1145/331499.331504

[B16] Kubota M. Hashiguchi T. (2020). Large-Scale Expression and Purification of Mumps Virus Hemagglutinin-Neuraminidase for Structural Analyses and Glycan-Binding Assays. Methods Mol. Biol. 2132, 641–652. 10.1007/978-1-0716-0430-4_55 32306363

[B17] Kubota M. Matsuoka R. Suzuki T. Yonekura K. Yanagi Y. Hashiguchi T. (2019). Molecular Mechanism of the Flexible Glycan Receptor Recognition by Mumps Virus. J. Virol. 93 (15), e00344–19. 10.1128/JVI.00344-19 31118251 PMC6639266

[B18] Kubota M. Takeuchi K. Watanabe S. Ohno S. Matsuoka R. Kohda D. (2016). Trisaccharide Containing α2,3-linked Sialic Acid Is a Receptor for Mumps Virus. Proc. Natl. Acad. Sci. USA. 113, 11579–11584. 10.1073/pnas.1608383113 27671656 PMC5068328

[B19] Lamb R. A. Kolakofsky D. (2001). “Paramyxoviridae: the Viruses and Their Replication,” in Fields Virology. Editors Knipe D. M. Howley P. M.. 4th ed. (Philadelphia: Lippincott, Williams & Wilkins).

[B20] Laskowski R. A. MacArthur M. W. Moss D. S. Thornton J. M. (1993). PROCHECK: a Program to Check the Stereochemical Quality of Protein Structures. J. Appl. Cryst. 26, 283–291. 10.1107/S0021889892009944

[B21] Morris G. M. Huey R. Lindstrom W. Sanner M. F. Belew R. K. Goodsell D. S. (2009). AutoDock4 and AutoDockTools4: Automated Docking with Selective Receptor Flexibility. J. Comput. Chem. 30, 2785–2791. 10.1002/jcc.21256 19399780 PMC2760638

[B30] Palacios G. Jabado O. Cisterna D. de Ory F. Renwick N. Echevarria J. E. (2005). Molecular Identification of Mumps Virus Genotypes from Clinical Samples: Standardized Method of Analysis. J. Clin. Microbiol. 43 (4), 1869–1878. 10.1128/JCM.43.4.1869-1878.2005 15815011 PMC1081370

[B22] Reeves P. J. Callewaert N. Contreras R. Khorana H. G. (2002). Structure and Function in Rhodopsin: High-Level Expression of Rhodopsin with Restricted and Homogeneous N-Glycosylation by a Tetracycline-Inducible N-Acetylglucosaminyltransferase I-Negative HEK293S Stable Mammalian Cell Line. Proc. Natl. Acad. Sci. 99 (21), 13419–13424. 10.1073/pnas.212519299 12370423 PMC129688

[B23] Ribeiro M. M. B. Franquelim H. G. Castanho M. A. R. B. Veiga A. S. (2008). Molecular interaction studies of peptides using steady-state fluorescence intensity. Static (de)quenching revisited. J. Pept. Sci. 14 (4), 401–406. 10.1002/psc.939 17994617

[B24] Schrodinger (2012). Epik Version 2.3, Impact version5.8, Prime Version 3.1. New York, NY: Schrodinger, LLC.

[B25] Schrödinger Release (2021). Schrödinger Release 2021-1: MacroModel. New York, NY: Schrödinger, LLC.

[B26] Schrödinger Suite (2019). Schrödinger Suite 2019-2 Protein Preparation Wizard; Epik, Impact, Prime. New York, NY: Schrödinger, LLC.

[B27] States D. J. Haberkorn R. A. Ruben D. J. (1982). A Two-Dimensional Nuclear Overhauser experiment with Pure Absorption Phase in Four Quadrants. J. Magn. Reson. (1969) 48, 286–292. 10.1016/0022-2364(82)90279-7

[B32] Turnbull W. B. Pease A. R. Stoddart J. F. (2000). Toward the Synthesis of Large Oligosaccharide-Based Dendrimers. ChemBioChem 1 (1), 70–74. 10.1002/1439-7633(20000703) 11828401

[B28] Waterhouse A. Bertoni M. Bienert S. Studer G. Tauriello G. Gumienny R. (2018). SWISS-MODEL: Homology Modelling of Protein Structures and Complexes. Nucleic Acids Res. 46 (W1), W296–W303. 10.1093/nar/gky427 29788355 PMC6030848

[B29] Willocks L. J. Guerendiain D. Austin H. I. Morrison K. E. Cameron R. L. Templeton K. E. (2017). An Outbreak of Mumps with Genetic Strain Variation in a Highly Vaccinated Student Population in Scotland. Epidemiol. Infect. 145 (15), 3219–3225. 10.1017/S0950268817002102 28903791 PMC9148756

[B33] Winger M. von Itzstein M. (2012). Exposing the Flexibility of Human Parainfluenza Virus Hemagglutinin-Neuraminidase. J. Am. Chem. Soc. 134 (44), 18447–18452. 10.1021/ja3084658 23057491

[B34] Yoneda Y. Kawada T. Rosenau T. Kosma P. (2005). Synthesis of Methyl 4’-O-Methyl-13C12-Beta-D-Cellobioside from 13C6-D-Glucose. Part 1: Reaction Optimization and Synthesis. Carbohydrate Research 340 (15), 2428–2435. 10.1016/j.carres.2005.08.003 16153619

