# Supplementary material for: Characterization of Natural and Synthetic Sialoglycans Targeting the Hemagglutinin-Neuraminidase of Mumps Virus
Source: Front Chem. 2021 Oct 27;9:711346. doi: 10.3389/fchem.2021.711346 (PMC8578797; doi:10.3389/fchem.2021.711346)
Supplement: Supplementary file 1 [file DataSheet1.docx]

**Supporting Material**

**Characterization of natural and synthetic sialoglycans targeting the Hemagglutinin-Neuraminidase of mumps virus**

Rosa Ester Forgione^1^, Cristina Di Carluccio^1^, Francesco Milanesi^2,3^, Marie Kubota^4^, Ferran Fabregat Nieto^1^, Antonio Molinaro^1^, Takao Hashiguchi^5^, Oscar Francesconi^2^, Roberta Marchetti^1^*, Alba Silipo^1^

1 Department of Chemical Sciences, Complesso Universitario Monte Sant’Angelo, University of Naples Federico II, Via Cintia 4, I-80126, Napoli, Italy.

2 Department of Chemistry "Ugo Schiff" and INSTM, University of Florence Polo Scientifico e Tecnologico, 50019, Sesto Fiorentino, Firenze, Italy.

3 Magnetic Resonance Center CERM, Via L. Sacconi 6, I–50019 Sesto Fiorentino, Firenze, (Italy)

4 Department of Virology, Faculty of Medicine, Kyushu University, Fukuoka, 812-8582, Japan.

5 Laboratory of Medical Virology, Department of Virus Research, Institute for Frontier Life and Medical Sciences, Kyoto University, Kyoto 606-8507, Japan

**Supporting Tables:** Tables S1-S2 pag S2

**Supporting Figures:** Figures S1-S14 pag S3-S12

**Supporting Tables**

**Table S1.** **^1^H and ^13^C chemical shifts (ppm) of 3’SLN (Neu5Ac-α-(2,3)-Gal-β-(1,4)-GlcNAc-β-O(CH_2_)_2_NH_2_).**

| **Unit** | **1** | **2** | **3** | **4** | **5** | **6** | **7** | **8** | **9** |
| --- | --- | --- | --- | --- | --- | --- | --- | --- | --- |
| GlcNAcA residue |  |  |  |  |  |  |  |  |  |
| ^1^H | 4.52 | 3.77 | 3.73 | 3.73 | 3.61 | 4.02\|3.86 |  |  |  |
| ^13^C | 101.18 | 55.09 | 72.16 | 78.28 | 74.78 | 60.00 |  |  |  |
| **CH_3_** |  |  |  |  |  |  |  |  |  |
| ^1^H |  | 1.96 |  |  |  |  |  |  |  |
| ^13^C |  | 26.76 |  |  |  |  |  |  |  |
| **Gal**  **B residue** |  |  |  |  |  |  |  |  |  |
| ^1^H | 4.56 | 3.58 | 4.12 | 3.97 | 3.72 | 3.74\|3.73 |  |  |  |
| ^13^C | 102.54 | 69.41 | 75.53 | 67.46 | 75.26 | 61.01 |  |  |  |
|  |  |  |  |  |  |  |  |  |  |
| **Neu5Ac**  **K residue** |  |  |  |  |  |  |  |  |  |
| ^1^H |  |  | Eq\|Ax  2.77\|1.81 | 3.70 | 3.85 | 3.65 | 3.61 | 3.90 | 3.88\|3.66 |
| ^13^C |  |  | 39.81 | 68.46 | 51.66 | 72.93 | 68.18 | 71.84 | 62.69 |
| **CH_3_** |  |  |  |  |  |  |  |  |  |
| ^1^H |  |  |  |  | 1.95 |  |  |  |  |
| ^13^C |  |  |  |  | 26.76 |  |  |  |  |

**Table S2.** **^1^H and ^13^C chemical shifts (ppm) of thio-3’SL (Neu5Ac-α-(2,3)-Gal-β-(1,4)-Glc-β-OCH_3_).**

| **Unit** | **1** | **2** | **3** | **4** | **5** | **6** | **7** | **8** | **9** |
| --- | --- | --- | --- | --- | --- | --- | --- | --- | --- |
| GlcA residue |  |  |  |  |  |  |  |  |  |
| ^1^H | 4.42 | 3.32 | 3.52 | 3.60 | 3.56 | 4.03\|3.86 |  |  |  |
| ^13^C | 103.16 | 72.74 | 74.84 | 78.04 | 74.36 | 60.00 |  |  |  |
|  |  |  |  |  |  |  |  |  |  |
| **Gal**  **B residue** |  |  |  |  |  |  |  |  |  |
| ^1^H | 4.55 | 3.40 | 3.38 | 3.88 | 3.69 | 3.74\|3.72 |  |  |  |
| ^13^C | 104.13 | 68.22 | 50.58 | 68.74 | 77.75 | 61.01 |  |  |  |
|  |  |  |  |  |  |  |  |  |  |
| **Neu5Ac**  **K residue** |  |  |  |  |  |  |  |  |  |
| ^1^H |  |  | Eq\|Ax  2.82\|1.83 | 3.68 | 3.87 | 3.59 | 3.59 | 3.94 | 3.88\|3.65 |
| ^13^C |  |  | 40.38 | 68.46 | 51.49 | 74.88 | 68.06 | 71.95 | 62.69 |
| **CH_3_** |  |  |  |  |  |  |  |  |  |
| ^1^H |  |  |  |  | 2.04 |  |  |  |  |
| ^13^C |  |  |  |  | 21.90 |  |  |  |  |

**Supporting Figures**

**Figure S1. ESI-MS spectrum (positive ion mode) of compound 4 (direct injection of a 10** **μg/mL solution in MeOH)**

**Figure S2. ESI-MS spectrum (negative ion mode) of compound 1 (direct injection of a 10 μg/mL solution in H_2_O)**

**Figure S3. ^1^H NMR spectrum of compound 4 (500 MHz, CDCl3).**

**
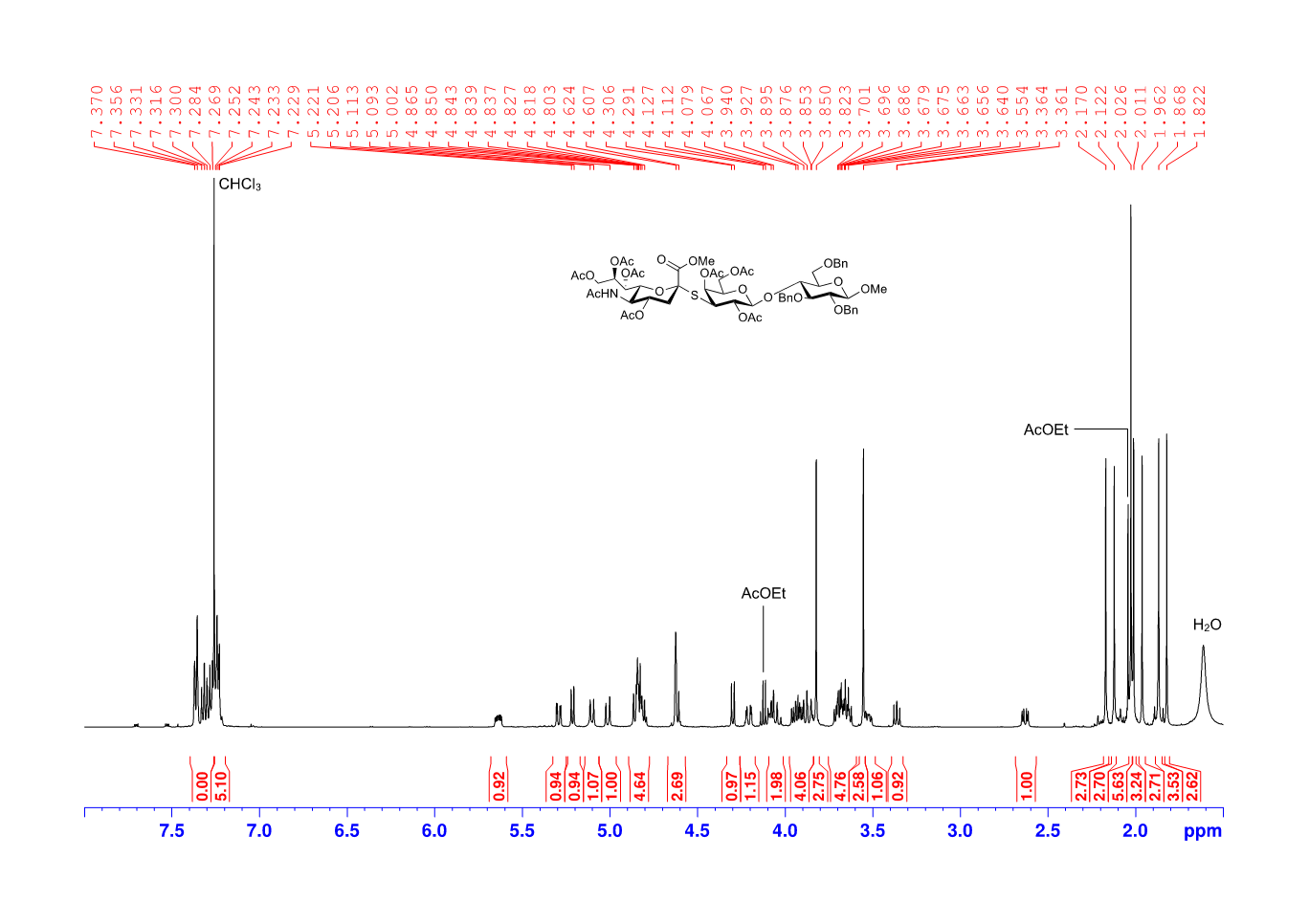
**

**Figure S4: ^13^C NMR spectrum of compound 4 (125 MHz, CDCl3).**

**
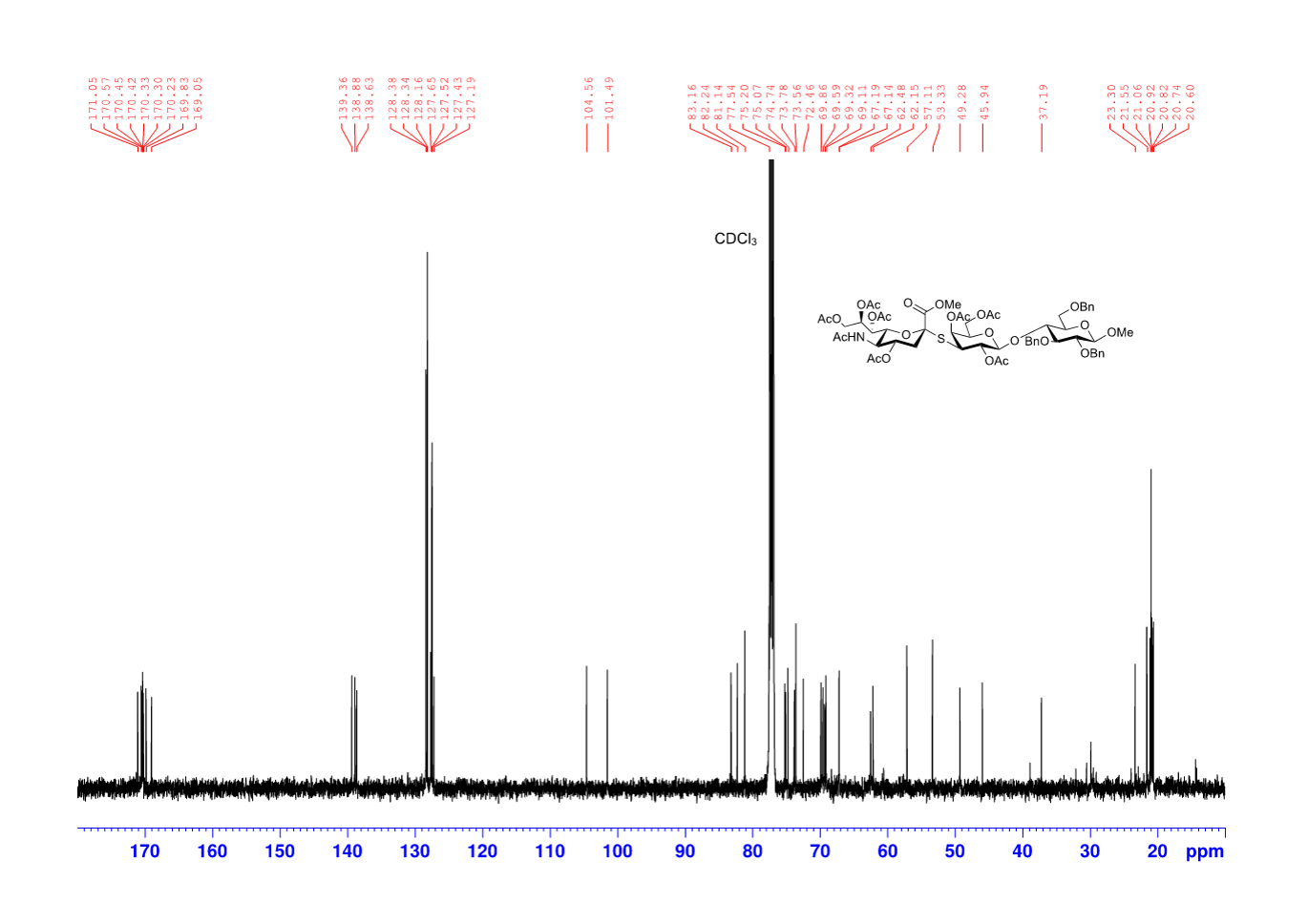
**

**Figure S5. COSY spectrum of compound 4 (500 MHz, CDCl3)**

**Figure S6. ^1^H NMR spectrum of compound 1 (500 MHz, D2O).**

**
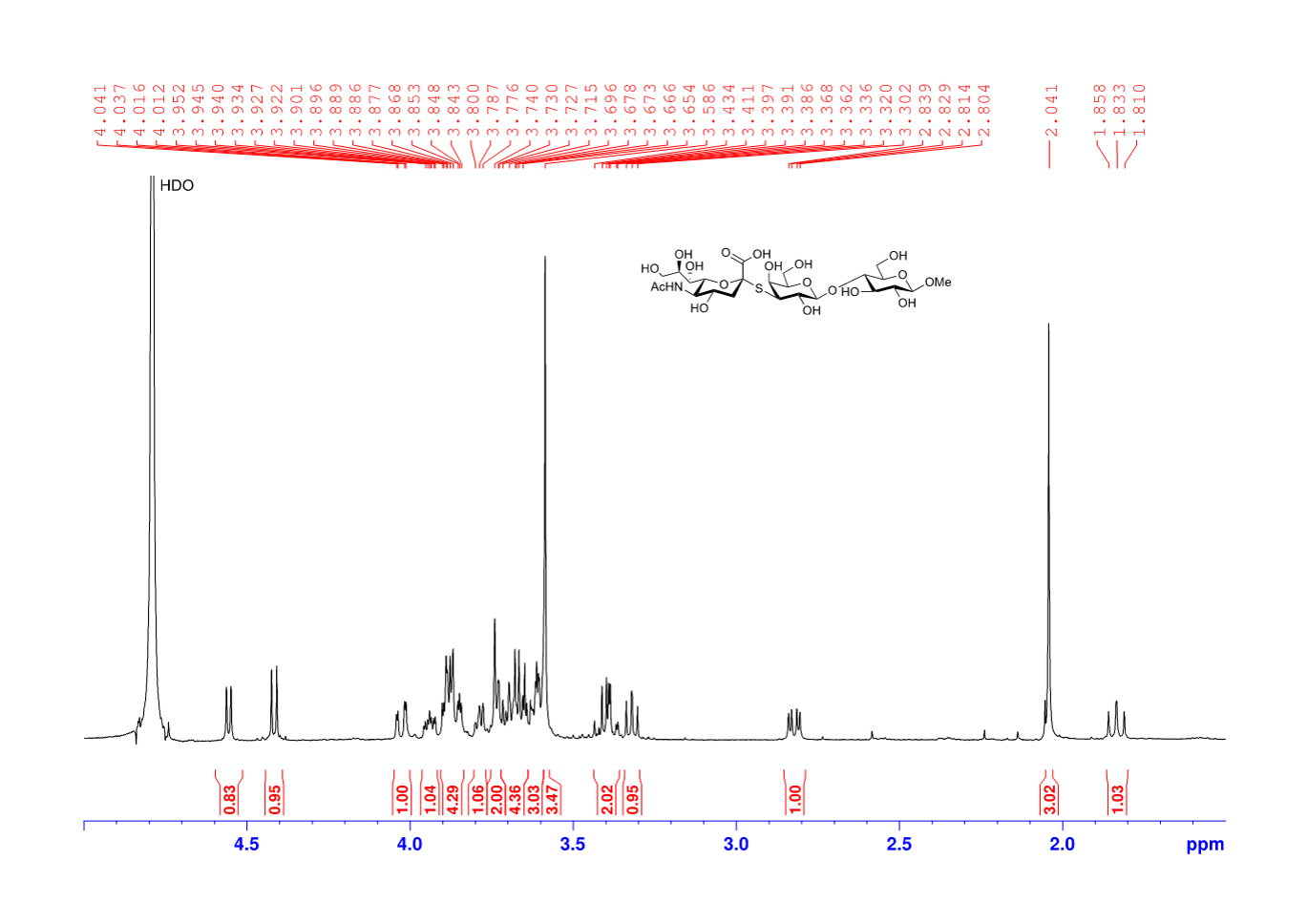
**

**Figure S7. ^13^C NMR spectrum of compound 1 (125 MHz, D2O).**

**
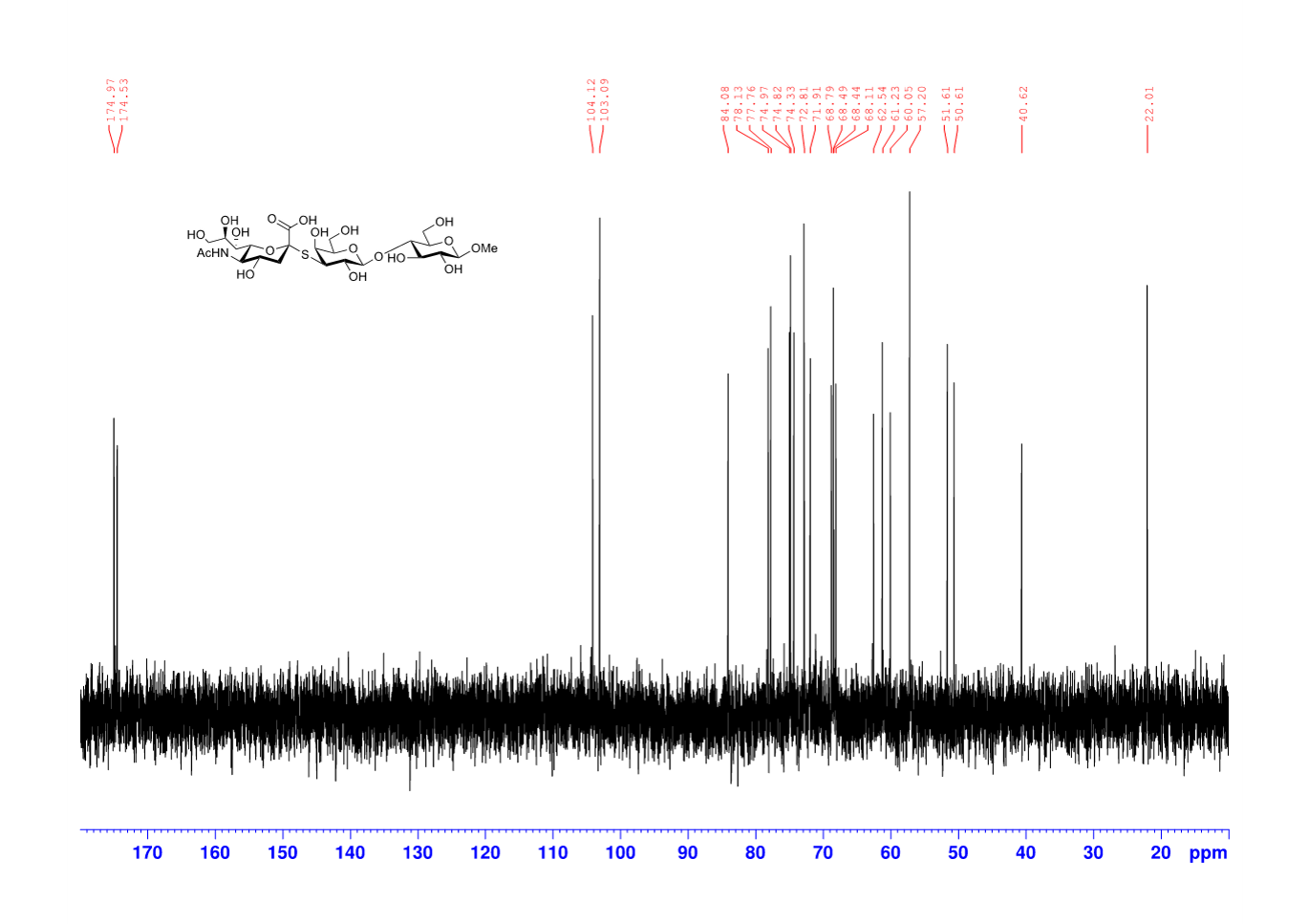
 Figure S8. COSY spectrum of compound 1 (500 MHz, CDCl3)**

**Figure S9. ^1^H-^13^C HSQC spectrum of 3’SLN (Neu5Ac-α-(2,3)-Gal-β-(1,4)-GlcNAc-β-O(CH_2_)_2_NH_2_).**

**
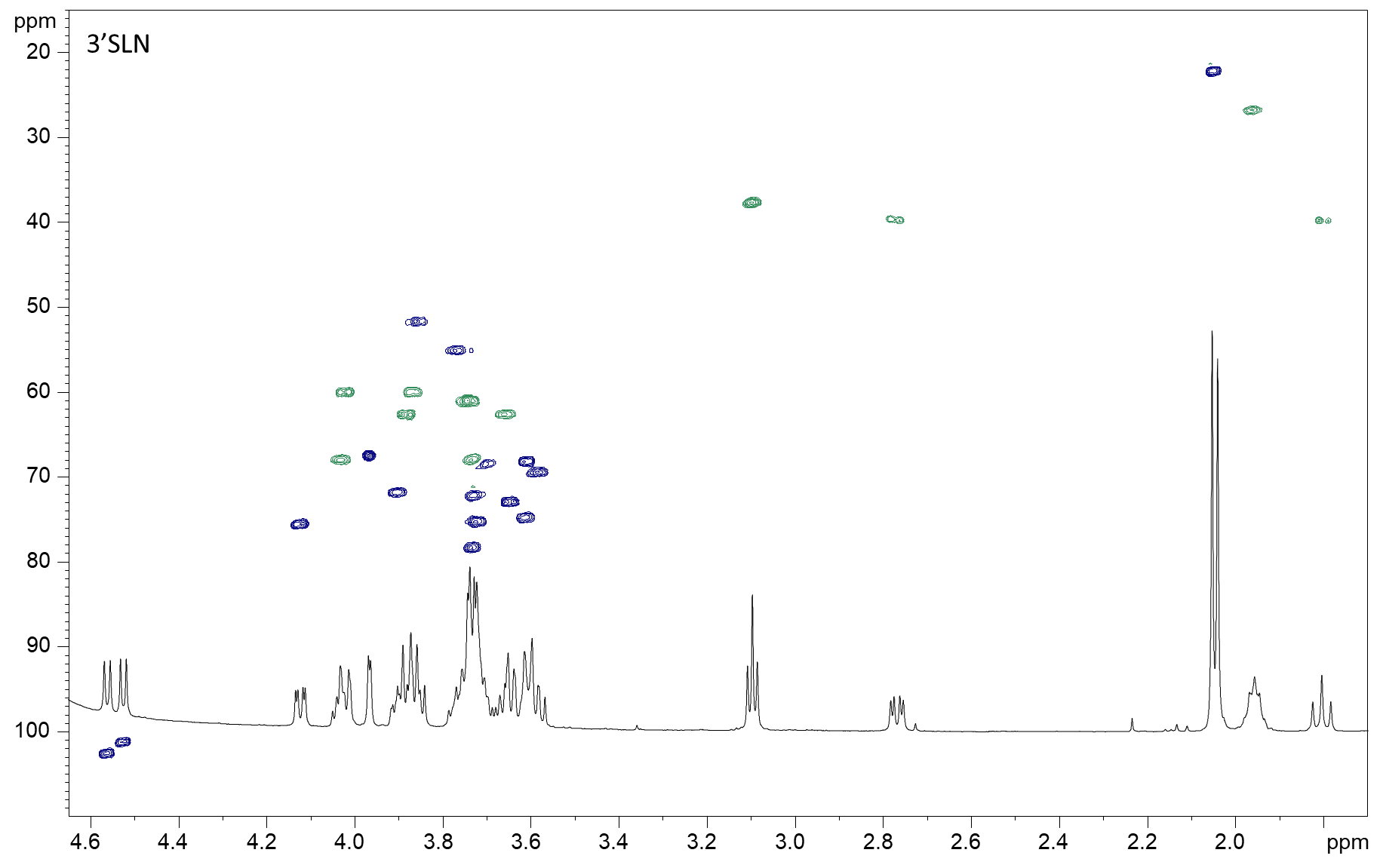
**

**Figure S10. ^1^H-^13^C HSQC spectrum of thio-3’SL ^1^H and ^13^C chemical shifts (ppm) of thio-3’SL (Neu5Ac-α-(2,3)-Gal-β-(1,4)-Glc-β-OCH_3_).**

**
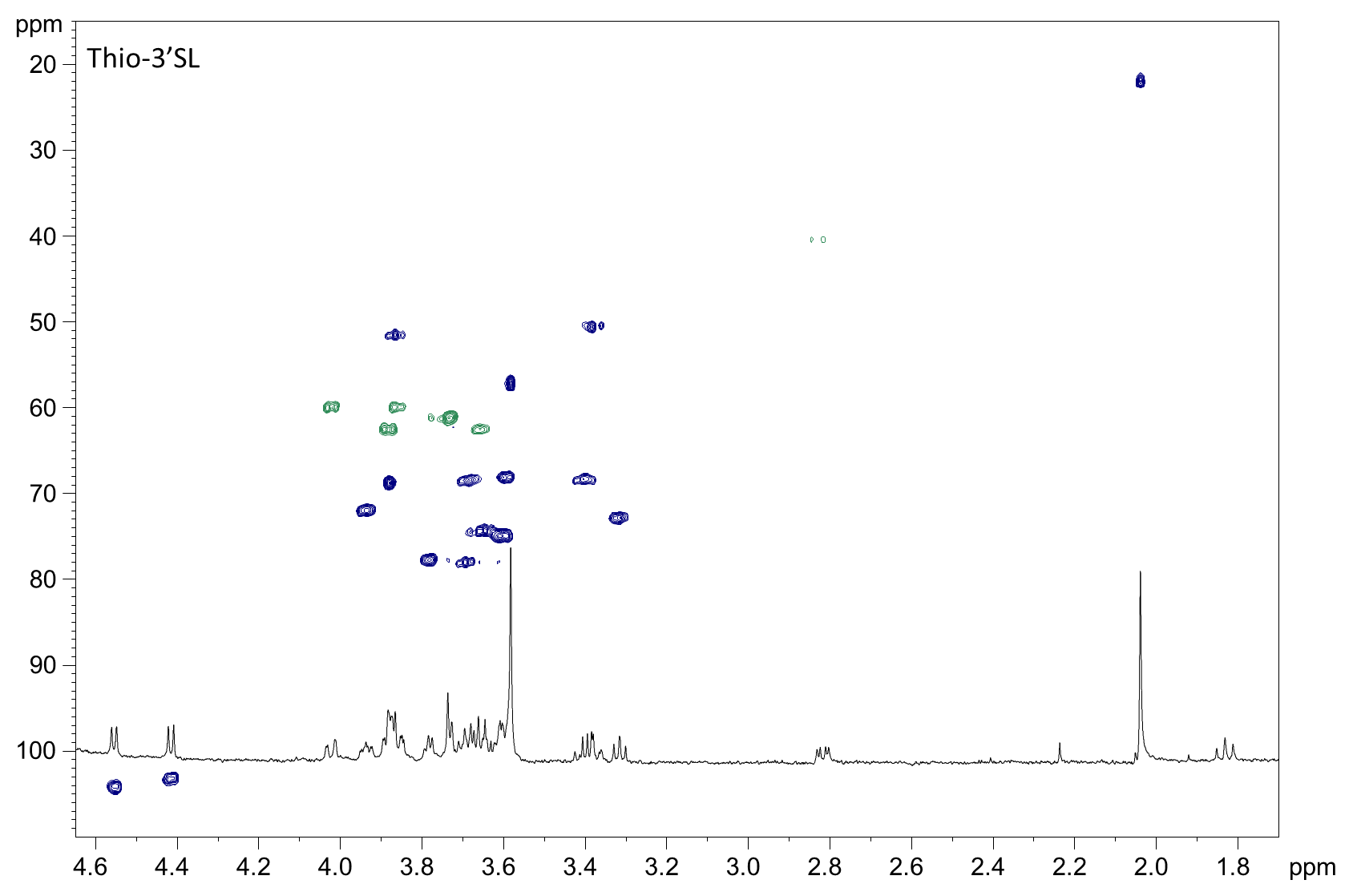
**

**Figure S11. Validation of the homology model of MuVHN-SBL-1 a)** Superimposition of the head domains of MuV-HN SBL-1 model (green), and MuV-HN Hoshino strain crystal structure, PDB entry 5B2D (blue). b) PROCHECK analysis (Ramachandran plot) **c)** Verify3D plot of the homology model.


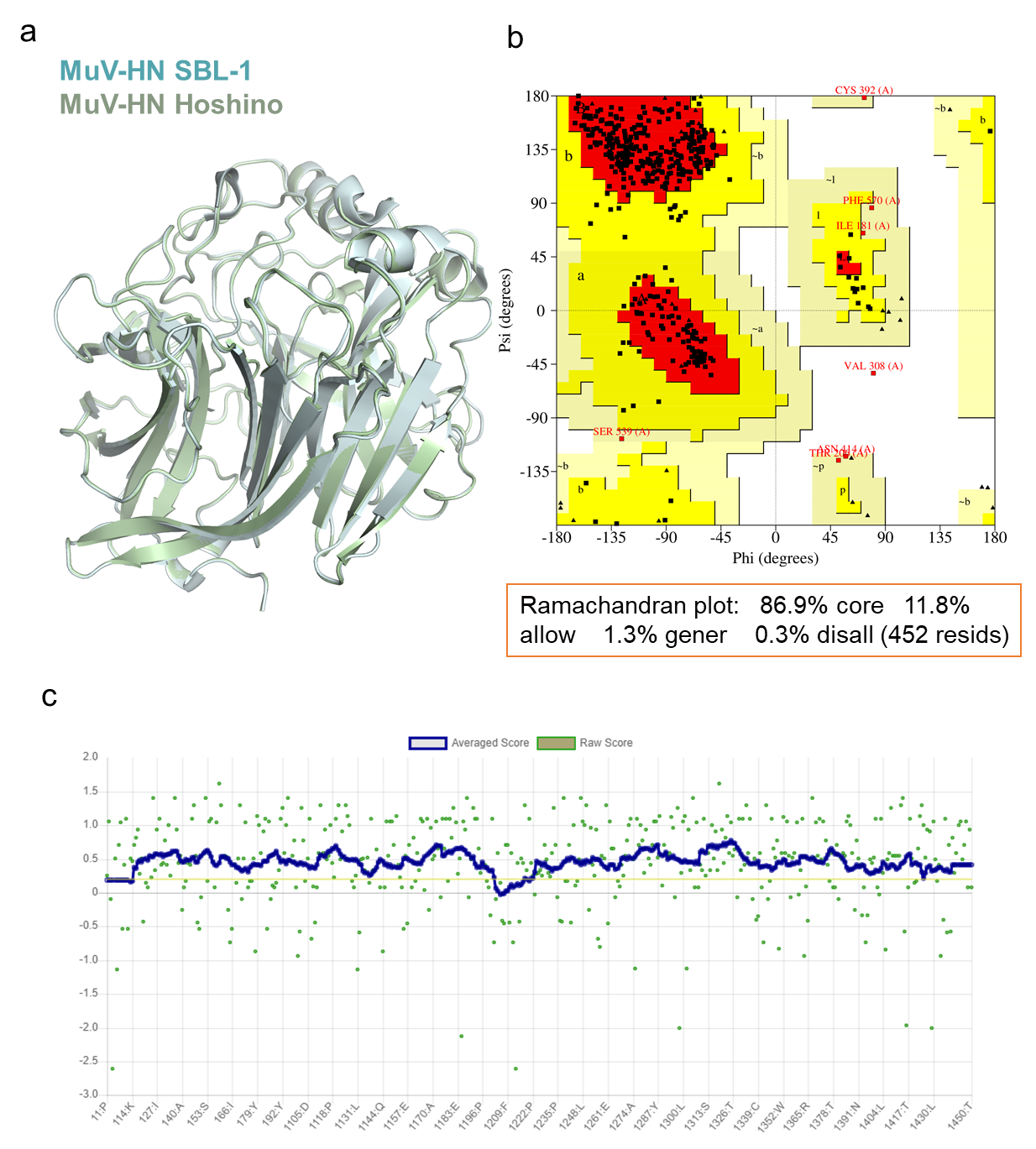


**Figure S12. MD simulation analysis of MuV-HN homology model. a)** Backbone RMSD of the protein along the trajectory. **b)** Backbone RMSD of the inter-strand loops, namely ß1, ß2, ß3, ß4, ß5, ß6.**c)** Per residue atomic fluctuation of the structure, calculated using the protein Cα atoms. The peaks in the RMSF plot corresponded to the mobile loops connecting the six ß-sheets and the β strands in each of the β-sheets. Inter-strand loops: ß1 (Asn199-Asn209), ß2 (Val259-Gln276), ß3 (Tyr365-Val375), ß4 (Thr438-Ser446), ß5 (Phe501-Ile515), ß6 (Glu561-Phe570) Inter-sheet loops: β2-β3 (Ile291-Asn300), ß4-ß5 (Ile452-Ser477), β5-β6 (Phe530- Pro535).

**Figure S13 MD simulation analysis of MuV-HN/3’SLN complex. a)** Protein and ligand backbone RMSD variation along the MD. **b) S**uperimposition of the most populated clusters (C1-C4) derived according to k-mean algorithm estimation. **c)** Distance between MuV-HN triarginyl cluster: Arg180, Arg422 and Arg512 and Sia carboxylate. The average between the heavy atoms directly involved in the interaction has been considered **d)** Average distance between Tyr369 aromatic ring protons and H1-H3-H5 atoms of GlcNAc unit **e)** Frequency most representative protein/ligand distances.


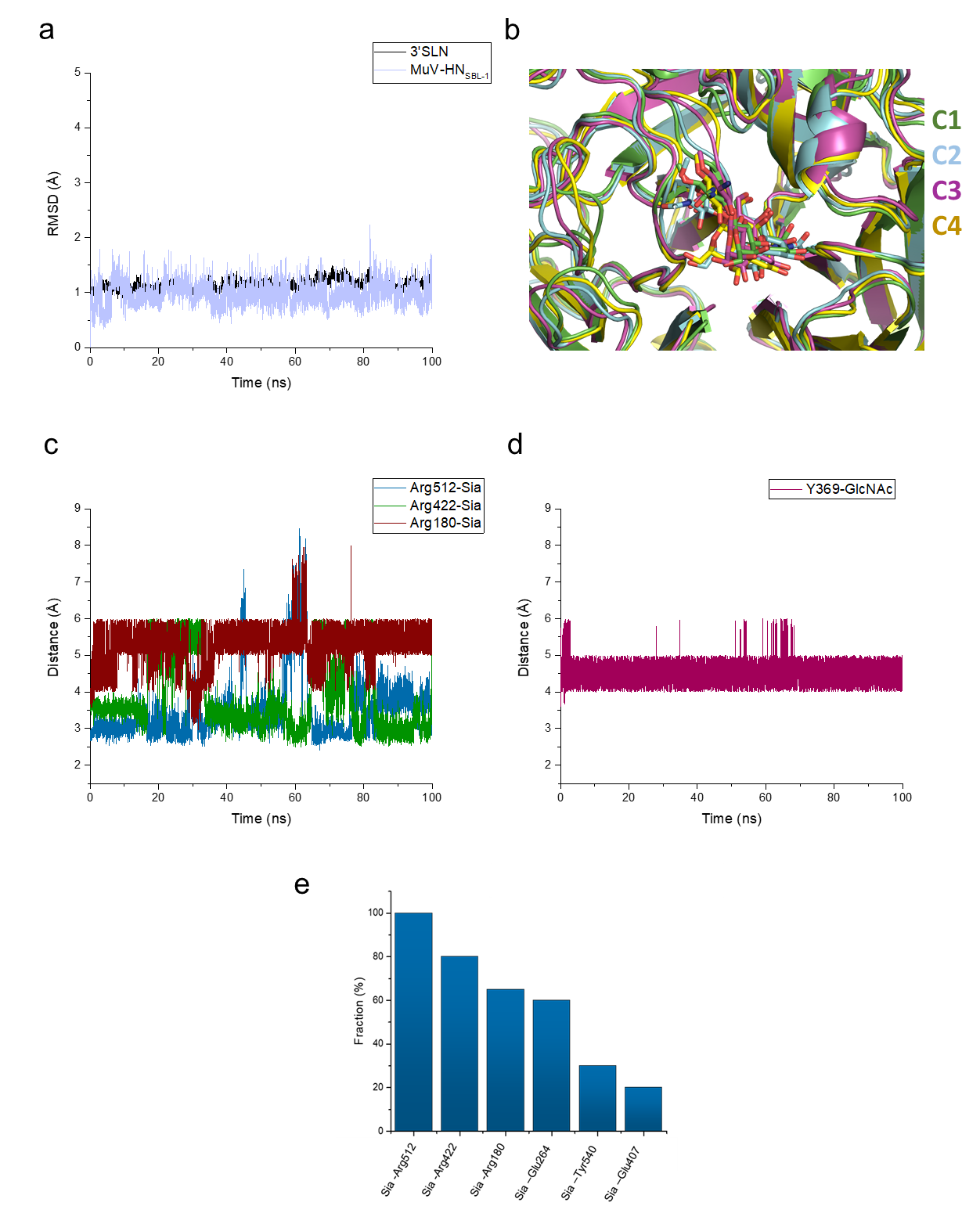


**Figure S14** **Flexibility of MuV-HN upon 3’SLN binding. a)** Superimposition of the Cα RMSF plots of apo-MuV-HN (black) and MuV-HN/3’SLN complex (red). The inter-sheet loops were indicated in blue and the inter-strand loops in red. Inter-strand loops: ß1 (Asn199-Asn209), ß2 (Val259-Gln276), ß3 (Tyr365-Val375), ß4 (Thr438-Ser446), ß5 (Phe501-Ile515), ß6 (Glu561-Phe570) Inter-sheet loops: β2-β3 (Ile291-Asn300), ß4-ß5 (Ile452-Ser477), β5-β6 (Phe530- Pro535). **b)** Backbone RMSD of MuV-HN inter-strand loops in the complex, namely ß1, ß2, ß3, ß4, ß5 and ß6.
